# Convex approaches to isolate the shared and distinct genetic components of complex traits

**DOI:** 10.1093/bioinformatics/btag670

**Published:** 2026-09-17

**Authors:** Saikat Banerjee, Shane O’Connell, Sarah M C Colbert, Niamh Mullins, David A Knowles

**Affiliations:** New York Genome Center, New York, NY 10013, United States; Department of Psychiatry, Icahn School of Medicine at Mount Sinai, New York, NY 10029, United States; Department of Genetics and Genomic Sciences, Icahn School of Medicine at Mount Sinai, New York, NY 10029, United States; Charles Bronfman Institute for Personalized Medicine, Icahn School of Medicine at Mount Sinai, New York, NY 10029, United States; Department of Artificial Intelligence and Human Health, Icahn School of Medicine at Mount Sinai, New York, NY 10029, United States; Department of Psychiatry, Icahn School of Medicine at Mount Sinai, New York, NY 10029, United States; Department of Genetics and Genomic Sciences, Icahn School of Medicine at Mount Sinai, New York, NY 10029, United States; Charles Bronfman Institute for Personalized Medicine, Icahn School of Medicine at Mount Sinai, New York, NY 10029, United States; Department of Artificial Intelligence and Human Health, Icahn School of Medicine at Mount Sinai, New York, NY 10029, United States; Department of Psychiatry, Icahn School of Medicine at Mount Sinai, New York, NY 10029, United States; Department of Genetics and Genomic Sciences, Icahn School of Medicine at Mount Sinai, New York, NY 10029, United States; Charles Bronfman Institute for Personalized Medicine, Icahn School of Medicine at Mount Sinai, New York, NY 10029, United States; Department of Artificial Intelligence and Human Health, Icahn School of Medicine at Mount Sinai, New York, NY 10029, United States; New York Genome Center, New York, NY 10013, United States; Department of Computer Science, Columbia University, New York, NY 10027, United States; Department of Systems Biology, Columbia University, New York, NY 10032, United States

## Abstract

**Motivation:**

Groups of complex diseases, such as coronary heart disease, neuropsychiatric disorders, and cancers, often display overlapping clinical symptoms and pharmacological responses. Genetic variants with shared associations across diseases have the potential to help explain their underlying biological processes, but this sharing remains poorly understood.

**Results:**

We model the matrix of summary statistics of trait-associated genetic variants as the sum of a low-rank component—representing shared biological processes—and a sparse component representing disease-unique processes and arbitrarily corrupted or contaminated components. We introduce Clorinn, an open-source Python library that uses convex optimization algorithms to recover these components by minimizing a weighted combination of nuclear norm and L1 terms. Clorinn provides two significant benefits: (i) convex optimization guarantees reproducibility of the components and (ii) the low-rank “uncorrupted” matrix allows robust singular value decomposition and principal component analysis, which are otherwise highly sensitive to outliers and noise in the input matrix. In extensive simulations, we observe that Clorinn is uniquely able to recover the disease-group structure while remaining competitive on factor-level reconstruction error. We apply Clorinn to estimate 200 latent factors from GWAS summary statistics for 2110 phenotypes from the Pan-UK Biobank (*N* = 420 531 European-ancestry individuals) and 10 latent factors from 14 psychiatric disorders.

**Availability and implementation:**

Clorinn is available at https://github.com/daklab/clorinn.

## 1 Introduction

The genetic architecture of complex traits is governed by the interplay of shared and distinct influences. Shared influences are shaped by pleiotropy (Solovieff *et al.* 2013), wherein single genetic variants exert effects across multiple biological processes. Genome-wide association studies (GWAS) have identified thousands of genetic variant–trait associations, revealing that numerous variants are associated with a diverse array of traits—spanning psychiatric conditions to metabolic syndromes—in patterns that often elude straightforward clinical categorization (Bulik-Sullivan *et al.* 2015, Tanigawa *et al.* 2019). The emergence of large biobank datasets with thousands of phenotypes (e.g. UK Biobank) facilitates comprehensive, phenome-wide analyses of genetic overlap and specificity. Dissecting this polygenic architecture is fundamental to elucidating the biological etiology of complex traits and refining therapeutic interventions. Yet, disentangling the genetic factors common to multiple traits from those uniquely attributable to specific phenotypes, amidst the confounding, noise, and high dimensionality inherent in genomic datasets, poses a formidable analytical challenge.

Various methods have been developed to characterize the joint genetic architecture of traits. Bivariate causal mixture models (MiXeR) (Frei *et al.* 2019) quantify the degree of variant effect overlap between pairs of traits, but they do not output variant-specific parameters and cannot jointly model more than a few traits. Even a trivariate extension (Shadrin *et al.* 2024) still cannot model higher-dimensional pleiotropy. Genomic structural equation modeling (gSEM) (Grotzinger *et al.* 2019) extends genetic correlation analysis by fitting user-specified factor models to the genetic covariance matrix of traits. While powerful for testing hypotheses, gSEM requires that one prespecify the number of latent factors and their connectivity *a priori*, necessitating strong assumptions or prior knowledge about trait groupings. Misspecifying these can lead to unstable or biased results, yet choosing them correctly for any given set of complex traits is non-trivial.

More recent efforts have turned to data-driven dimensionality reduction of GWAS results (Tanigawa *et al.* 2019, Chasman *et al.* 2020, Zhang *et al.* 2023, Omdahl *et al.* 2025). For example, Decomposition of Genetic Associations applied truncated singular value decomposition (tSVD) to identify major components of genetic variation across thousands of phenotypes (Tanigawa *et al.* 2019). Similarly, Bayesian factor models have been introduced to infer latent pleiotropic factors from summary statistics. FactorGo, for instance, uses variational Bayesian factor analysis to infer pleiotropic factors from biobank-scale data (Zhang *et al.* 2023). Other methods have incorporated network or sparsity constraints [e.g. graphical lasso-based trait networks (Chung *et al.* 2017)] to cluster traits by shared associations (Omdahl *et al.* 2025). However, matrix factorization-based methods face several key challenges. First, approaches based on classical matrix factorization (such as PCA/SVD or standard factor analysis) are not robust to outliers or noise inherent in the data (Goodall 1988); even a single outlier in the data matrix can dominate the recovered factors. GWAS summary statistic matrices contain heterogeneous signal: some variant–trait associations are true pleiotropic hits, others are trait-specific, and some may be spurious (technical artifacts or stochastic noise). A single highly associated variant unique to one trait can act as an outlier that disproportionately skews the extracted factors in methods like SVD or Bayesian models assuming Gaussian noise. Yet most existing methods lack a mechanism to handle such contamination. Second, nearly all current factorization-based methods solve a non-convex optimization problem. Whether using expectation-maximization or variational Bayes, these algorithms can converge to different local optima depending on initial parameter seeds. In practice, this means solutions may vary greatly when applied to the same data or upon inclusion of extra traits or variants. Fixing a random seed does not fully alleviate this concern, as the addition of new data can lead to a different local minimum being found even if the objective landscape only changed slightly. Finally, flexibility often comes at the cost of computational complexity. Some advanced methods [e.g. pleiotropic decomposition regression (Ballard and O’Connor 2022)] have computational cost that scales poorly, taking on the order of hours to fit just a handful of traits. In summary, there is a pressing need for a method that can handle exploratory multi-trait analysis at scale while being robust to noise/outliers and yielding reproducible results.

To address these challenges, we introduce Clorinn (Convex LOw-Rank Inference via Nuclear Norm), a robust, scalable, and reproducible framework for decomposing GWAS summary statistic matrices. Clorinn models the *Z* score matrix as the sum of two components: a low-rank matrix capturing shared genetic signal across traits, and a sparse matrix representing trait-specific effects or corrupted entries (which may emanate from multiple sources of variation). Rather than relying on factorization of the raw data, Clorinn first estimates an “outlier-free” low-rank approximation via convex optimization, using nuclear norm minimization (NNM). This convex formulation ensures that the optimization objective contains no spurious local minima. Consequently, for fixed data and regularization parameters, the recovered low-rank solution is independent of initialization (up to numerical tolerance). Crucially, the inclusion of a sparse error term allows Clorinn to downweight or isolate outlier signals that could otherwise bias the decomposition. By separating the shared and distinct components of the genetic architecture, Clorinn offers a robust preprocessing step prior to downstream matrix factorization. Once the low-rank matrix is estimated, conventional tools like singular value decomposition (SVD) can be applied to extract latent factors. This modular strategy makes Clorinn adaptable to a wide range of multivariate GWAS analyses. Compared to previous matrix factorization methods, Clorinn introduces three key advances: (i) a convex formulation of the low-rank recovery problem, which yields solutions that are independent of initialization (for fixed data and tuning parameters) and facilitates stable interpretation across multiple runs or addition of new information; (ii) robustness through explicit modeling of sparse contamination, allowing entry-wise outliers to be separated from shared structure; and (iii) scalability, enabled by efficient implementations that accommodate thousands of traits and tens of thousands of SNPs. Together, these properties make Clorinn a principled and practical framework for large-scale investigations of shared genetic architecture and trait-specific divergence.

We demonstrate via extensive simulations that Clorinn is capable of recovering underlying disease-group structure with greater accuracy, in the presence of varying degrees of noise, than existing methods. Applied to 2110 real traits from the Pan-UK Biobank, Clorinn recovers trait clusters and disease category-specific factors consistent with published biology. Finally, we apply Clorinn to 14 psychiatric traits and characterize shared genetic latent factors to demonstrate its utility in a more focused setting.

## 2 Materials and methods

### 2.1 Method overview

We assume that the noisy matrix of observed *Z* scores for *N* traits and *P* SNPs, $Z\in R^{N\times P}$, can be decomposed as a sum of a low-rank matrix $X\in R^{N\times P}$ and some error $M\in R^{N\times P}$,


(1) Z=X+M.

X corresponds to the shared genetic components of the traits. The problem to recover X from noisy Z is called low-rank matrix approximation (LRMA). It seeks the best rank-*k* estimate of X by solving the problem,


$$\begin{array}{cc} \mathrm{minimize} & {\left\|Z-X\right\|}_{F}\\ \text{subject}\;\text{to} & \text{rank}\;\left(X\right)\leq K. \end{array}$$

where ${\left\|\cdot \right\|}_{F}$ denotes the Frobenius norm. There are two popular approaches for LRMA: low-rank matrix factorization (LRMF) and rank minimization.

LRMF seeks to recover latent structures in the data by factorizing the original data matrix Z into a linear combination of *K* shared trait-specific loadings $L\in R^{N\times K}$ with latent factors $F\in R^{P\times K}$,


(2) $$Z=LF^{⊤}+M.$$

It uses the estimates $\hat{L}$ and $\hat{F}$ to reconstruct the low-rank estimate $\hat{X}≃\hat{L}{\hat{F}}^{⊤}$. Intuitively, the hidden factors F are some unknown underlying latent embeddings of the variants in *K* dimensions. These hidden factors can capture shared biological processes or pathways that drive complex diseases. The factorization in (2) assumes that factor–trait and variant–factor relationships are linear. The number of factors (or dimensions) *K* needed for faithful representation of Z will depend on the complexity of the data. As in classical principal component analysis (PCA) (Hotelling 1933, Eckart and Young 1936, Goodall 1988), (2) can be efficiently solved via SVD if L and F are unregularized and the noise M is small and *i.i.d.* Gaussian. Other common strategies, such as independent components analysis and non-negative matrix factorization enforce desired properties on L and F. Several new LRMF approaches, including tSVD (Tanigawa *et al.* 2019) and FactorGo (Zhang *et al.* 2023), have been developed specifically for learning latent factors underlying GWAS traits. tSVD applies SVD and truncates the columns of L and F to obtain low rank. FactorGo uses Bayesian priors on L and F to enforce sparsity. However, these methods are susceptible to the shortcomings mentioned above, in particular:

**Outliers and noise.** One major weakness of classical PCA is its brittleness with respect to grossly corrupted or outlying observations (Goodall 1988): A single grossly corrupted entry in M could render the estimated $\hat{X}$ arbitrarily far from the true X. These methods assume that the noise is Gaussian, i.e. M∼N(0,Σ) is a matrix of Gaussian residuals with variance Σ. In many cases, particularly for heterogeneous diseases, the *Z* scores may have non-Gaussian outliers and discrepancies

**Non-convex optimization.** Many matrix factorization models are formulated as non-convex optimization problems. Such methods may depend on initialization and can converge to different local optima in practice. Many practitioners fix the random seeds, which is only a bandaid solution as tiny perturbations in the data may yield very different results. Classical PCA has a closed-form global solution via SVD under Gaussian noise assumptions. However, it is sensitive to gross entry-wise outliers, which can substantially distort principal components.

In this article, we focus on rank minimization methods to obtain a low-rank, “outlier-free” matrix X, before obtaining the matrix factorization via SVD. In the context of computer vision and compressed sensing, a class of robust PCA methods has been developed to handle outliers and high noise (Ke and Kanade 2005, Wright *et al.* 2009, Xu *et al.* 2010, Candès *et al.* 2011, Bouwmans and Zahzah 2014). Following these methods, we assume that the errors M can be arbitrary in magnitude but sparsely supported, affecting only a fraction of the entries of Z. The outliers and discrepancies captured by the sparse component M may include: (a) the private contribution of a few key variants to specific phenotypes or (b) errors in the available summary statistics due to, e.g. allele flipping, imputation errors, or meta-analysis artifacts.

Therefore, in place of Gaussian noise, we assume that M is sparse for the rank minimization problem. Since direct rank minimization is itself a non-convex problem, it is often approximated by nuclear norm minimization: minimizing the nuclear norm ${\left\|X\right\|}_{*}$ (i.e. the sum of singular values) of the estimated matrix X, which is a convex relaxation of the matrix rank (Fazel 2002, Cai *et al.* 2010). NNM approximates Z by X, while constraining ${\left\|X\right\|}_{*}$. NNM is analogous to Lasso regression which approximates minimizing the number of non-zero regression coefficients by instead minimizing the $\ell_{1}$ norm (sum of absolute values) of the coefficients.

Here, we use different convex optimization algorithms to perform LRMA:

1. Robust PCA (RPCA) using inexact alternating Lagrangian multipliers (IALM) algorithm,
2. NNM using Frank–Wolfe (FW) algorithm, and its variant,
3. Sparse NNM (NNM-Sparse) using FW algorithm.

We use Algorithm 5 from Lin *et al.* (2010) with an improved approach for adapting the step size for RPCA and derive novel FW algorithms for NNM (see Section 2.2).

Having estimated $\hat{X}$ via one of these methods, we use tSVD to obtain $\hat{L}$ and $\hat{F}$ (Fig. 1). Our convex algorithms guarantee reproducible estimates of $\hat{X}$. By applying tSVD on $\hat{X}$ instead of Z, we avoid the shortcomings of classical PCA and obtain more robust estimates of the latent factors. In a statistical genetics context, this is especially pertinent; technical noise, measurement error, and population structure can have subtle impacts on association study test statistics. Characterization of latent components representing shared genetic effects via PCA is sensitive to variation arising from these non-biological sources. The estimation of $\hat{X}$ facilitates the description of accurate and reproducible latent genetic factors robust to the presence of outliers, allowing us to mitigate these issues.

**Figure 1:**
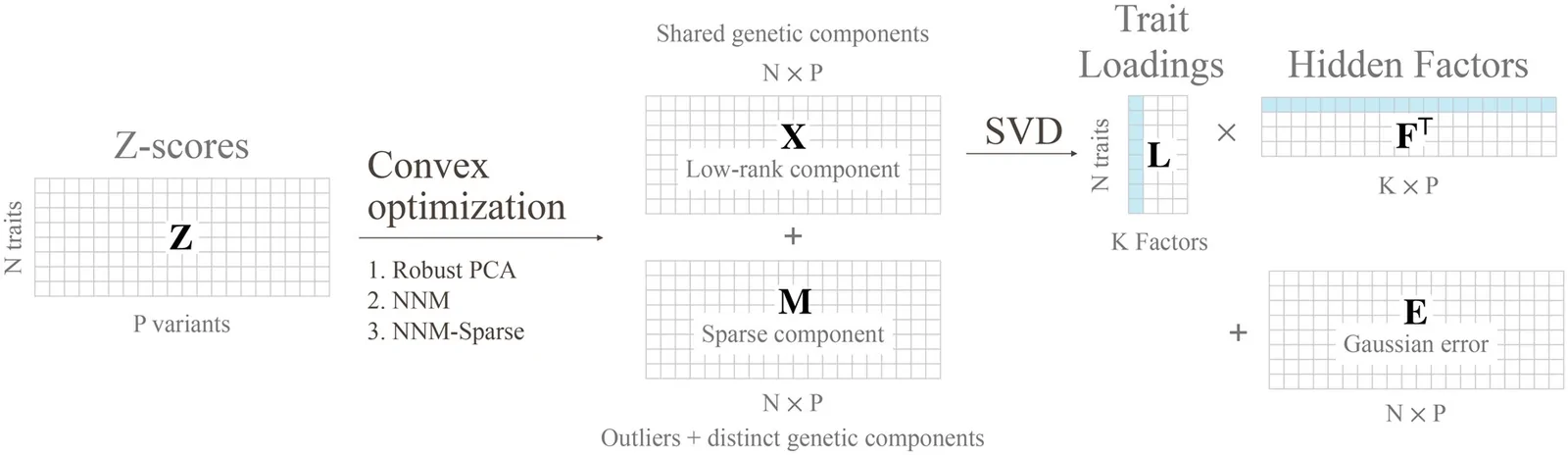
Schematic representation of Clorinn.

### 2.2 Frank–Wolfe for rank minimization

We use the FW algorithm (Frank and Wolfe 1956, Jaggi 2013, Ding and Udell 2018) to solve the constrained optimization problem,


(3) $$\underset{X}{\text{min}}\;\frac{1}{2}{\left\|Z-X\right\|}_{F}^{2}\;\text{subject}\;\text{to}\;{\left\|X\right\|}_{*}\leq r.$$

Most methods (Yu and Kong 2025) for solving NNM, including singular value thresholding (Cai *et al.* 2010) and proximal methods (Toh and Yun 2010), are computationally expensive since they require solving the full SVD each iteration with cost $O(\text{min}(NP^{2},N^{2}P))$, limiting scalability. FW uses significantly less expensive linear subproblems per iteration since it requires only an approximate leading eigenvector. For example, FW type techniques have been successfully used to develop fast algorithms to solve structural SVMs (Lacoste-Julien *et al.* 2013), and NNM problems (Jaggi and Sulovský 2010). For the FW algorithm, the loss function for the NNM problem is,


(4) $$f\left(X\right)=\frac{1}{2}\sum_{i}\sum_{j}{\left(z_{ij}-x_{ij}\right)}^{2},$$

and its gradient is,


(5) ∇f(X)=X−Z.

We outline the steps for FW in Algorithm 1. The main bottleneck in implementing the algorithm is solving the linear optimization subproblem in Line 2. The objective of the subproblem is linear even though the constraints in the set Ω may not be. Since Ω is compact, the solution to the subproblem always exists. After finding a solution $S_{t}$ to the linear optimization subproblem, Line 8 finds an appropriate step size to move in the direction $D_{t}:=X_{t-1}-S_{t}$. The new iterate is a convex combination of the previous iterate and $S_{t}$.

Algorithm 1. Frank–Wolfe algorithm for nuclear norm minimization

**Input:** *f*, ∇f, *r*

**Initialize:**  $X_{0}$ such that ${\left\|X_{0}\right\|}_{*}\leq r$

1: **for**  t=1,2,…,T  **do** 

2:  $S_{t}=\underset{S\;\in \;\Omega }{\arg\;\min}\;\mathrm{Tr}\left(\nabla f{\left(X_{t-1}\right)}^{⊤}S\right)$,

    where $\Omega =\left\{S\in R^{N\times P}|{\left\|S\right\|}_{*}\leq r\right\}$

3:  $D_{t}:=X_{t-1}-S_{t}$

4:  $g_{t}=\mathrm{Tr}\left(D_{t}^{⊤}\nabla f\left(X_{t-1}\right)\right)$

5:  **if**  $g_{t}\leq \epsilon$  **then** 

6:   **break** 

7:  **end if** 

8:  $\gamma_{t}=\frac{2}{2+t}$ or $\gamma_{t}=\underset{\gamma }{\arg\;\min}\;f\left(X_{t-1}-\gamma D_{t}\right)$

9:  $X_{t}=X_{t-1}-\gamma_{t}D_{t}$

10: **end for** 

The term $g_{t}$ is the surrogate duality gap which is a certificate of the current approximation quality, i.e. $g_{t}\geq f(X_{t-1})-f(\hat{X})$, where $\hat{X}$ is the solution of the optimization. Although $\hat{X}$ is not known, we can easily compute $g_{t}$, which can be used as a convergence criterion.

#### 2.2.1 Linear optimization subproblem

Denote the SVD of $\nabla f\left(X_{t-1}\right)$ as $\mathrm{UD}V^{⊤}$, where $U\in R^{N\times K}$ and $V\in R^{P\times K}$ are orthogonal matrices, $U^{⊤}U=V^{⊤}V=I_{K}$. The columns of U and the columns of V are called left-singular vectors and right-singular vectors, respectively. $D\in R^{K\times K}$ is a diagonal matrix whose diagonal entries are the singular values. The rank of the matrix is at most min{N,P}. Then the solution to the subproblem in Line 2 is the convex hull of the singular vectors with maximal singular value, appropriately scaled. In practice, we take the solution $S_{t}=-ru_{1}v_{1}^{⊤}$, where $u_{1}$ and $v_{1}$ denote the first columns of U and V, respectively (Jaggi 2013, Ding and Udell 2018).

#### 2.2.2 Step size

The optimal step size for the subproblem in Line 8 can be derived analytically. Define


(6) $$X^{*}:=X_{t-1}-\gamma D_{t}.$$

From (4), we have,


(7) $$f\left(X^{*}\right)=\frac{1}{2}\sum_{i}\sum_{j}{\left(z_{ij}-x_{ij}^{*}\right)}^{2}$$

The derivative of this function is,


(8) $$\begin{array}{c} \frac{\partial }{\partial \gamma }f\left(X^{*}\right)=\sum_{i,j}\left(z_{ij}-x_{ij}^{*}\right)d_{ij}\\ =\sum_{i,j}\left(z_{ij}-x_{ij}\right)d_{ij}+\gamma \sum_{i,j}d_{ij}^{2}\\ =-\mathrm{Tr}\left(D_{t}^{⊤}\nabla f\left(X_{t-1}\right)\right)+\gamma {\left\|D_{t}\right\|}_{F}^{2}\\ =-g_{t}+\gamma {\left\|D_{t}\right\|}_{F}^{2}. \end{array}$$

Setting this equal to 0, we obtain the optimum step size,


(9) $$\gamma_{t}=\frac{g_{t}}{{\left\|D_{t}\right\|}_{F}^{2}}.$$

The NNM algorithm is benchmarked against the softImpute algorithm (Hastie *et al.* 2014) in Fig. S1, available as supplementary data at *Bioinformatics* online.

### 2.2.3 NNM-Sparse

As discussed in Section 2.1, we want to extract sparse outliers from the input data. We propose the joint optimization problem over X and M,


(10) $${\text{min}}_{X,M}\;\frac{1}{2}{\left\|Z-X-M\right\|}_{F}^{2}\;\text{subject}\;\text{to}\;{\left\|X\right\|}_{*}\leq r,{\left\|M\right\|}_{1}\leq l.$$

which we use FW to solve (Algorithm 2). The loss function and the gradients are given by,


(11) $$\begin{array}{c} f\left(X,M\right)=\frac{1}{2}\sum_{i}\sum_{j}{\left(z_{ij}-x_{ij}-m_{ij}\right)}^{2},\\ \nabla_{X}f=\nabla_{M}f=X+M-Z. \end{array}$$

Here, we need to solve two linear optimization subproblems, corresponding to the two different constraints. The nuclear norm regularization for the subproblem in Line 2 is exactly the same as in Algorithm 1. The $\ell_{1}$ norm regularization for the subproblem in Line 3 can be solved by noting that the dual of the $\ell_{1}$ norm is the $\ell_{\infty }$ norm, i.e. ${\left\|Y\right\|}_{\infty }:={\text{max}}_{\left(i,j\right)}\left|y_{ij}\right|={\text{max}}_{{\left\|Q\right\|}_{1}\leq 1}\mathrm{Tr}\left(Y^{⊤}Q\right)$. The optimization problem ${\text{min}}_{Q}\mathrm{Tr}\left(Y^{⊤}Q\right)$ such that ${\left\|Q\right\|}_{1}\leq 1$ has optimal value $-{\left\|Y\right\|}_{\infty }$. Therefore, one minimizer for Line 3 is $S_{M}=-l\cdot \mathrm{sign}\left({\left[\nabla_{M}f\right]}_{i*j*}\right)\;e_{i*}e_{j*}^{⊤}$ where $\left(i*,j*\right)\in \arg\;\max_{i,j}\;\left|x_{ij}+m_{ij}-z_{ij}\right|$, i.e. $S_{M}$ has exactly one non-zero element. In the standard FW algorithm, the update for the sparse component updates only one element of the matrix (Line 11). To improve the speed of the algorithm, we include an additional step within each loop to minimize wrt the sparse component, as suggested by Mu *et al.* (2016). Given a threshold β>0, the projection onto the $\ell_{1}$ ball minimizes the sparse component,


(12) $$P_{{\left\|\cdot \right\|}_{1}\leq \beta }\left[Y\right]=\underset{{\left\|Q\right\|}_{1}\leq \beta }{\arg\;\min}\;\frac{1}{2}{\left\|Y-Q\right\|}_{F}^{2}.$$

As shown previously (Mu *et al.* 2016), the projected gradient step in Line 12 ensures that the objective is less than that produced by the standard FW step, i.e. $f\left(X_{t},M_{t}\right)\leq f\left(X_{t},M_{t-\frac{1}{2}}\right)$.

Algorithm 2. Frank–Wolfe algorithm for sparse nuclear norm minimization (10)

**Input:**  f(X,M), gradient functions $\left(\nabla_{X}f,\nabla_{M}f\right)$, constraints *r*, *l*

**Initialize:**  $X_{0}$, $M_{0}$ such that ${\left\|X_{0}\right\|}_{*}\leq r$, ${\left\|M_{0}\right\|}_{1}\leq l$

1: **for**  t=1,2,…,T  **do** 

2:  $S_{X}=\underset{S\;\in \;\Omega_{X}}{\arg\;\min}\;\mathrm{Tr}\left(\nabla_{X}f{\left(X_{t-1}\right)}^{⊤}S\right)$,

where $\Omega_{X}=\left\{S\in R^{N\times P}|{\left\|S\right\|}_{*}\leq r\right\}$

3:  $S_{M}=\underset{S_{M}\;\in \;\Omega_{M}}{\arg\;\min}\;\mathrm{Tr}\left(\nabla_{M}f{\left(M_{t-1}\right)}^{⊤}S_{M}\right)$,

where $\Omega_{M}=\left\{S_{M}\in R^{N\times P}|{\left\|S_{M}\right\|}_{1}\leq l\right\}$

4:  $D_{t}:=\left(D_{X},D_{M}\right)$,

where $D_{X}:=X_{t-1}-S_{X}$ and $D_{M}:=M_{t-1}-S_{M}$

5:  $g_{t}=\mathrm{Tr}\left(D_{t}^{⊤}\nabla f\left(X_{t-1},M_{t-1}\right)\right)$

6:  **if**  $g_{t}\leq \epsilon$  **then** 

7:  **break** 

8:  **end if** 

9:  $\begin{array}{l} \gamma_{t}=\frac{2}{2+t}\;\text{or}\\ \gamma_{t}=\underset{\gamma }{\arg\;\min}\;f\left(X_{t-1}-\gamma D_{X},M_{t-1}-\gamma D_{M}\right) \end{array}$

10:  $X_{t}=X_{t-1}-\gamma_{t}D_{X}$

11:  $M_{t-\frac{1}{2}}=M_{t-1}-\gamma_{t}D_{M}$

12:  $M_{t}=P_{{\left\|\cdot \right\|}_{1}\leq l}\left[M_{t-\frac{1}{2}}-\nabla_{M}f\left(X_{t},M_{t-\frac{1}{2}}\right)\right]$

13: **end for** 

### 2.3 Tuning the nuclear norm constraint

To determine the optimal nuclear norm constraint parameter *r* for Clorinn, we implemented a five-fold cross-validation strategy. For each fold, we randomly held out a subset of 16% observed entries from the GWAS *Z* score matrix as missing values for model validation, while another independent subset of 20% entries was reserved as a held-out test set for final evaluation. The subset of masked entries was independent in all folds for consistency. For each candidate *r*, Clorinn was trained to complete the matrix using NNM, and the masked entries were predicted. We evaluated performance using root mean squared error (RMSE), averaged across the five folds. The value of *r* that minimized validation RMSE was selected for the analysis. Finally, we retrained the model on the entire available training data (all entries except the held-out test set) using this optimal *r*, and evaluated its performance on the independent test set. Prediction error on the test set was assessed with the same metrics (RMSE) to confirm that the selected *r* generalizes well to completely unseen data. This cross-validation approach ensured that the nuclear norm constraint was tuned to optimally capture true pleiotropic signal in the *Z* score matrix while avoiding overfitting to noise, thus guiding the selection of an *r* that yields robust, accurate matrix completion results.

### 2.4 Simulation of shared traits

We performed simulations under the model of Equation (2). We first generate *K* orthonormal vectors (Mezzadri 2007) of length *P* (number of variants), which form the columns of $F\in R^{P\times K}$ and represent the hidden factors. Each column of F (denoted by $F_{:k}$) is a unit vector with mean 0, standard deviation $1/\sqrt{P}$ and is orthogonal to all other columns. For each hidden factor, we generate the latent embeddings for *N* studies (traits), which form the columns of $L\in R^{N\times K}$. To mimic the shared component of traits in real data, we sample each column of L from a multivariate normal distribution $L_{:k}∼N\left(0_{N},\Sigma \right)$ with $\Sigma =\left(gg^{⊤}⊙A\right)/K$, where ⊙ denotes element-wise multiplication. Here, $g\in R^{N}$ is a column vector with elements $g_{n}$. The total SNP heritability of the *n*th trait is $h_{n}^{2}$; such that $g_{n}^{2}$ is part of the SNP heritability contributed by the low-rank component $LF^{⊤}$, and $\left(h_{n}^{2}-g_{n}^{2}\right)$ is part of the SNP heritability contributed by the sparse component M. A is a block-diagonal matrix which encodes the strength of sharing between different traits within and across disease categories. Each block can be considered as representing a broad disease category with multiple traits. Let *Q* be the number of disease categories (number of blocks) in A and $A_{q}$ be the set of indices for the *q*th block. Then, each element $A_{ij}$ is given by,


(13) $$A_{ij}=\{\begin{array}{cc} 1, & \text{if}\;i=j,\\ \alpha_{q}, & \text{if}\;i\neq j\;\text{and}\;i,j\in A_{q},\\ \alpha_{0}, & \text{otherwise}. \end{array}$$

Here, $\alpha_{q}\in \left(0,1\right)$ is the amount of sharing between the traits of the *q*th disease category, whereas $\alpha_{0}\in \left(0,1\right)$ specifies the amount of sharing between the different disease categories.

We then generate $M\in R^{N\times P}$ by sampling each column $M_{:p}$ from a Laplace distribution,


(14) $$M_{:p}∼\mathrm{Laplace}\left(0_{N},\lambda \right),\;\lambda =\sqrt{\frac{h^{⊤}h-g^{⊤}g}{2P}}$$

to ensure that the total heritability for the traits is $h^{⊤}h$. Each column of M is the portion of the effect size of a SNP which cannot be explained by shared factors. The true effect sizes are then calculated as,


(15) $$Y=LF^{⊤}+M.$$

The maximum likelihood estimate for the variance of each observation is given by


(16) $${\hat{\sigma }}_{nj}^{2}=\frac{1-y_{nj}^{2}}{\nu_{n}},$$

where $\nu_{n}$ is the sample size of the *n*th study/trait, and assuming that the genotypes and phenotypes have been standardized. The observed effect sizes are then sampled from a normal distribution,


(17) $${\tilde{y}}_{nj}=N\left(y_{nj},{\hat{\sigma }}_{nj}^{2}\right)$$

Finally, we obtain the observed *Z* scores as $Z=\tilde{Y}/\hat{\sigma }$. For each simulation experiment, we used the default hyperparameters K=10, N=200, P=2000, and $h^{2}=0.2$. We then varied each parameter while holding all others fixed (e.g. we varied $h^{2}$ while keeping *K*, *N*, and *P* fixed at the default values above).

### 2.5 Characterization of hidden factors

The loadings and factors are obtained by SVD of the low-rank matrix $X=\mathrm{UD}V^{⊤}$. We define the orthonormal factors as F:=V and L:=UD. The loadings can also be interpreted as a projection of X on the factors,


$$L=\mathrm{UD}=\mathrm{UD}V^{⊤}V=\mathrm{XF}.$$

#### 2.5.1 Squared cosine score

The squared cosine score quantifies the importance of a factor *k* for trait *j* as,


(18) $${\;\text{cos}\;}_{k,j}^{2}=\frac{\ell_{j,k}^{2}}{\sum_{k^{\prime }}\ell_{j,k^{\prime }}^{2}},$$

where $\ell_{j,k}$ is the (j,k) entry of L. Factors with higher squared cosine scores contribute more to the total distance from the origin and are therefore more important for that trait. We used these scores to rank factors for each focal trait.

#### 2.5.2 Contribution score

Two complementary contribution scores measure how much a given trait or variant contributes to a focal factor. The trait contribution score is


(19) $${\text{contr}}_{k,j}^{\text{trait}}=\frac{\ell_{j,k}^{2}}{\sum_{j^{\prime }}\ell_{j^{\prime },k}^{2}},$$

and the variant contribution score is


(20) $${\text{contr}}_{k,p}^{\text{variant}}=\frac{f_{p,k}^{2}}{\sum_{p^{\prime }}f_{p^{\prime },k}^{2}},$$

where $f_{p,k}$ is the (p,k) entry of F. To interpret a factor, we focus on traits or variants with contribution scores greater than their respective uniform averages (1/N and 1/P).

### 2.6 Method comparison

Let $\hat{L}$ and $\hat{F}$ denote the estimated loadings and factors from the different methods applied on the simulated data. The factorization $\hat{X}=\hat{L}{\hat{F}}^{⊤}$ is not unique—it is invariant under any orthogonal rotation of $\hat{L}$ and $\hat{F}$, or any product-preserving column rescaling between them. We therefore apply a global amplitude calibration to $\hat{X}$, followed by Procrustes rotation and per-factor rescaling of $\hat{L}$ and $\hat{F}$, before comparing them with the ground truth.

#### 2.6.1 Global calibration

Different methods report $\hat{L}{\hat{F}}^{⊤}$ on systematically different scales. For example, flashier is fitted directly to the *Z* score input $Z=\tilde{Y}/\hat{\sigma }$, so its reconstruction lives at the *Z* score scale—a factor of $\sqrt{\nu }$ above the effect-size scale. FactorGo ingests the sample sizes and returns a reconstruction on the effect-size scale directly. Clorinn and tSVD apply an internal $1/\sqrt{NP}$ normalization before the SVD step. These per-method conventions are independent of how well each method recovers the latent structure, but if left uncorrected they dominate any factor-level error metric.

To isolate the scale mismatch from errors in recovered structure, we calibrated the estimated $\hat{X}$ by the least-squares scalar


(21) $$\hat{s}=\underset{s}{\arg\;\min}\;||X-s\hat{X}|{|}_{F}^{2}=\frac{{\left\langle X,\hat{X}\right\rangle }_{F}}{||\hat{X}|{|}_{F}^{2}},$$

where ${\langle A,B\rangle }_{F}=\mathrm{Tr}\left(A^{⊤}B\right)$ and $\hat{s}=0$ if $||\hat{X}|{|}_{F}=0$. We set ${\hat{X}}_{\text{cal}}=\hat{s}\hat{X}$. For factor-level comparisons, we assigned this scalar to the loadings ${\hat{L}}_{\text{cal}}=\hat{s}\hat{L}$ while $\hat{F}$ was left unchanged. Since $\hat{s}$ is a single global scalar, it leaves row- and column-wise structure unchanged. Methods that recover the correct structure with a globally miscalibrated amplitude are therefore not penalized, while methods with incorrect relative structure still are. We retain $\hat{s}$ as a diagnostic of the per-method scale convention (Fig. S2, available as supplementary data at *Bioinformatics* online). In what follows, we drop the subscript and refer to the calibrated estimates as $\hat{X}$ and $\hat{L}$ unless otherwise noted.

#### 2.6.2 Procrustes alignment

We then estimated the optimal rotation $\hat{R}$ by solving the orthogonal Procrustes problem (Gower 1975)


(22) $$\hat{R}=\underset{R}{\text{arg}\;\text{min}}\;||F-\hat{F}R|{|}_{F}^{2}\;\text{subject to}\;R^{⊤}R$$

and set $\tilde{F}=\hat{F}\hat{R}$ and $\tilde{L}=\hat{L}\hat{R}$. When the true and estimated factorizations had different numbers of factors, we first matched their latent dimensions by zero-padding the smaller. After rotation, we corrected residual per-factor scale differences by estimating a scalar for each factor *j*,


(23) $${\hat{d}}_{j}=\{\begin{array}{cc} 1, & \text{if}\;||{\tilde{f}}_{j}|{|}_{2}=0,\\ 1/||{\tilde{f}}_{j}|{|}_{2}, & \text{if}\;f_{j}=0\;\text{and}\;||{\tilde{f}}_{j}|{|}_{2}>0,\\ \underset{d\in R}{\text{argmin}}||f_{j}-d\;{\tilde{f}}_{j}|{|}_{2}^{2}=\frac{f_{j}^{⊤}{\tilde{f}}_{j}}{||{\tilde{f}}_{j}|{|}_{2}^{2}}, & \text{otherwise}, \end{array}$$

where $||\cdot |{|}_{2}$ denotes the Euclidean norm. The first case avoids rescaling a factor with no estimated signal. The second case handles zero-padded true factors under rank misspecification. Since no least-squares scale is identifiable when the true factor is zero, we place the estimated factor on the same unit-norm convention used in the simulation and move the scale into $\hat{L}$. The third case is the least-squares fit for the nonzero true factors. With $D=\text{diag}({\hat{d}}_{1},\ldots ,{\hat{d}}_{K})$, the aligned estimates were


(24) $${\hat{F}}^{\;\text{align}}=\hat{F}\hat{R}D,$$

(25) $${\hat{L}}^{\;\text{align}}=\hat{L}\hat{R}D^{-1}.$$

This coupled transformation preserves the reconstructed signal, ${\hat{L}}^{\;\text{align}}{\left({\hat{F}}^{\;\text{align}}\right)}^{⊤}=\hat{L}{\hat{F}}^{⊤}$, while making the latent factor coordinates comparable across methods.

#### 2.6.3 Comparison metrics

We evaluated recovery along two complementary dimensions.

For latent disease structure, we used the native estimated loadings (no calibration or alignment), so the score reflects the intrinsic performance of each method without oracle information. We centered the loadings by row, computed pairwise Euclidean distances between rows, and clustered rows with average-linkage agglomerative hierarchical clustering. To assess robustness to clustering resolution, we clustered the rows into five classes—slightly misspecified relative to the three true disease groups. Adjusted mutual information (MI) between recovered and true group labels served as the metric.

For reconstruction accuracy and factor-level recovery, we compared calibrated and aligned $\hat{L}$, $\hat{F}$, and $\hat{X}$ with their ground-truth counterparts. To measure accuracy, we used root mean square error (RMSE) and relative RMSE (rRMSE). For any matrix A and its estimate $\hat{A}$, we define


(26) $$\text{RMSE}(\hat{A})={\left[\frac{1}{NP}\sum_{i,j}{\left(A_{ij}-{\hat{A}}_{ij}\right)}^{2}\right]}^{\frac{1}{2}}=\frac{1}{\sqrt{NP}}||A-\hat{A}|{|}_{F},$$

(27) $$\text{rRMSE}(\hat{A})=\frac{\text{RMSE}(\hat{A})}{\text{RMS}(A)}=\frac{||A-\hat{A}|{|}_{F}}{||A|{|}_{F}},$$

where $\text{RMS}(A)=||A|{|}_{F}/\sqrt{NP}$ is the root mean square of the elements of A. Thus, relative RMSE is equivalent to the relative Frobenius error. Since $\hat{R}$ and ${\hat{d}}_{j}$ are fit against the ground truth, RMSE and rRMSE are oracle-aligned, best-case measures of factor-level recovery. They can be small even when the native loadings would not recover the disease groups. We therefore treat Adjusted MI as our primary metric of structural recovery and the accuracy metrics as complementary factor-level diagnostics that quantifies how closely each method’s estimate can be rotated and rescaled onto the ground truth.

When varying *P*, we report rRMSE because total signal was fixed as *P* varied; so the metric is comparable across matrix dimensions (entry-wise RMSE of a zero estimate, e.g. shrinks with *P*, while rRMSE remains 1). When varying the true rank *K*, all methods estimated up to 10 factors. In that setting, we report RMSE, since rRMSE for $\hat{F}$ is sensitive to the zero-padding of F required under rank misspecification.

### 2.7 Application to PanUKB data

We used the publicly available summary statistics data provided by Zhang *et al.* (2023)—the quality-controlled *Z* score matrix consisted of 2483 traits with 51 399 variants, details of preprocessing can be found therein. However, we removed prescription traits because they were consistent outliers. We also selected only variants that were associated with at least one trait with *P* ≤ 5 × 10^−8^. The final *Z* score matrix consisted of 2110 traits with 51 368 variants. The explained variance ratio between $\hat{X}$ and Z was calculated by the ratio of the Frobenius norms of the respective centered matrices.

For downstream per-variant analyses of each factor, we placed the factor loadings on the input *Z* score scale following the pseudo-*Z* score construction of Zhang *et al.* (2023). For each factor *k*, we defined a pseudo sample size


(28) $$N_{k}^{\text{pseudo}}=\sum_{j=1}^{N}{\;\text{cos}\;}_{k,j}^{2}\nu_{j},$$

where $\nu_{j}$ is the sample size of the *j*th GWAS and ${\;\text{cos}\;}_{k,j}^{2}$ is the squared cosine score of factor *k* for trait *j* (Section 2.5). The per-variant pseudo-*Z* score for factor *k* is


(29) $$Z_{:k}^{\text{pseudo}}={\left(N_{k}^{\text{pseudo}}\right)}^{\frac{1}{2}}\;F_{:k}.$$

We used these pseudo-*Z* scores as the input for tissue-specific enrichment analysis.

### 2.8 Application to 14 psychiatric traits

We clumped SNPs per disorder using genotypes from 16 886 European individuals from the HRC as a reference with the following parameters in PLINK1.9: $P<5\times 10^{-5}$, window = 10 000 kb, $r^{2}<0.1$ (Purcell *et al.* 2007). For OUD summary statistics, we obtained the full panel of tested SNPs in European cohorts from the authors (Deak *et al.* 2022). We then annotated SNPs by position and allele using the 1000 Genomes European reference panel (SNP build 151) (The 1000 Genomes Project Consortium 2015). We filtered SNPs for missingness across phenotypes: SNPs not present in more than four phenotypes were excluded. We applied Clorinn with the NNM-sparse algorithm and five-fold cross-validation to estimate the optimal constraint parameter. We obtained hidden factors using tSVD on the shared output matrix.

We tested for a difference in means between numbers of traits where SNPs are significant across sets using an independent *t*-test of means. We derived a test statistic per SNP for association with PC values using the procedure described in Yamamoto *et al.* (2014). Briefly, we performed an SVD of the input X matrix to obtain the right-singular vectors $V^{⊤}$. The PC score is the product of the input data multiplied by the right-singular vectors, yielding a N×N matrix (where *N* is the number of traits). The association score was obtained by regressing the scaled values of the PC from this matrix across phenotypes on the SNP association *Z* scores across phenotypes. We repeated this operation for every SNP and used FUMA (Watanabe *et al.* 2017) to carry out functional enrichment analysis. We used SNPNexus to map SNPs to their nearest genes, and used ShinyGO version 0.82 to carry out enrichment analysis. We used FUMA for post-association analyses and plotted gene-based association test statistic results using a custom Python script.

We ran RPCA using a λ value of 0.016, which was chosen to yield a M matrix with approximately 50% nonzero values.

To more rigorously assess statistical significance for the number of traits where a SNP was statistically significant across “pleiotropic” and non-pleiotropic SNP sets, we resampled 3120 SNPs 1000 times from Z and reran independent *t*-tests. We found no test statistics more extreme than our observed results in these resamplings.

## 3 Results

### 3.1 Simulation experiments

We characterize how well Clorinn is able to recover the underlying hidden factors under different conditions and compare its performance to related approaches using simulated data. We simulated summary statistics from Equation (2) (see Section 2.4) for 200 traits representing three disease categories (e.g. in Fig. S3a, available as supplementary data at *Bioinformatics* online). The heritability of each trait ranged between 5% and 40%, half of which comes from the shared factors and the rest comes from the sparse, unique variants. The simulated hidden factors were orthonormal, and their loadings were sampled from a multivariate Gaussian distribution with a block-diagonal covariance matrix. As a result, the squared Pearson correlation ($r^{2}$) between trait rows of L exhibits a block-diagonal structure (Fig. 2a), with traits within a disease category more strongly correlated than traits across different categories. The loadings of the factors would clearly distinguish the different heterogeneous disease groups (Fig. S3a, available as supplementary data at *Bioinformatics* online). However, neither the “true” *Z* scores nor the loadings are observed in the simulated data. Our task is to recover the “true” loadings from the noisy simulated data.

**Figure 2:**
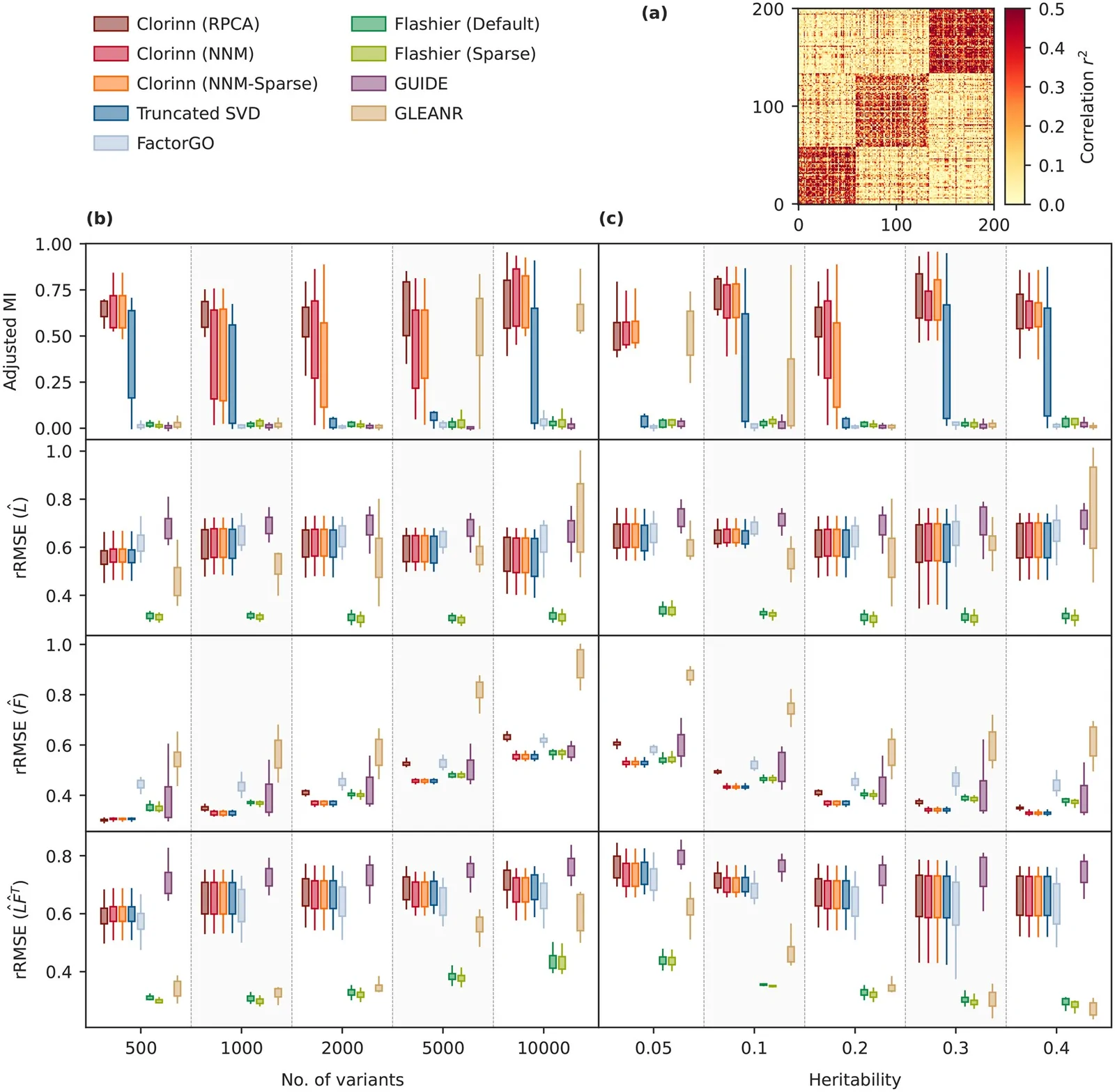
Clorinn accurately classifies subtypes of heterogeneous traits in realistic simulations. (a) Squared Pearson correlation ($r^{2}$) between trait rows of the ground-truth loading matrix L in one arbitrarily chosen simulation replicate (200 traits from three disease categories). The block-diagonal structure reflects stronger correlations within than across disease categories. (b) Comparison of methods as the number of variants *P* is varied, with the remaining parameters held at their default values. Each row reports one recovery metric, and each boxplot summarizes 20 simulation replicates. Adjusted MI is obtained by hierarchical clustering of each method’s *native* estimated loadings $\hat{L}$ (no calibration or alignment) and measures whether the recovered partition matches the true disease categories. $\text{rRMSE}(\hat{L})$ and $\text{rRMSE}(\hat{F})$ were obtained after global amplitude calibration and Procrustes alignment with per-factor rescaling against the ground truth. $\text{rRMSE}(\hat{L}{\hat{F}}^{⊤})$ is the relative RMSE of the reconstructed low-rank signal $\hat{X}=\hat{L}{\hat{F}}^{⊤}$ after global calibration; the alignment step is product-preserving and does not affect this metric. (c) Same as (b) but varying the per-trait heritability $h^{2}$, with P=2000 fixed.

We compare Clorinn with the five other methods, namely, tSVD (Tanigawa *et al.* 2019), FactorGo (Zhang *et al.* 2023), flashier (Willwerscheid *et al.* 2024), GUIDE (Lazarev *et al.* 2025), and GLEANR (Omdahl *et al.* 2025). For Clorinn, we first estimate the low-rank matrix $\hat{X}$ using the three different algorithms (RPCA, NNM, and NNM-Sparse) implemented in our software. We then perform the matrix factorization of $\hat{X}$ using tSVD. For all other methods, namely tSVD, FactorGo, flashier, GUIDE, and GLEANR, we perform the matrix factorization directly on the input matrix. For FactorGo, we used the default parameters. For flashier, we used the default Gaussian distribution for the prior on factors, combined with two different prior distributions for the loadings: (i) the default Gaussian prior, we call it “Flashier (Default)” and (ii) a point-normal prior, we call it “Flashier (Sparse)” as it encourages sparse loadings for the factors. We also used the most general possible variance structure allowed by flashier, estimating precision parameter for each row and column. GLEANR expects a matrix of estimated correlation due to sample sharing, for which we provided the covariance matrix of the observed *Z* scores, and allowed for Ledoit–Wolf shrinkage with parameter γ=0.5. We used the default parameters for GUIDE. Since it is challenging for any method to infer the number of latent factors, we specified the number of factors to be 10, which is the ground truth used for all simulations, except those where we vary the number of factors to evaluate model misspecification.

We evaluate each method along two complementary axes (see Section 2.6). For structural recovery, we report the adjusted MI (Vinh *et al.* 2010) between the true trait groups and groups recovered by clustering the native estimated loadings $\hat{L}$—our primary metric, as it reflects each method’s intrinsic ability to disentangle the disease groups without any oracle correction. For factor-level recovery, we report the relative RMSE of $\hat{L}$, $\hat{F}$, and $\hat{L}{\hat{F}}^{⊤}$ after global amplitude calibration and Procrustes alignment with per-factor rescaling against the ground truth.

We vary the number of variants (Fig. 2b) and the heritability (Fig. 2c). Across all settings, Clorinn attains the highest adjusted MI, recovering the three disease groups from its native loadings without any oracle rotation or rescaling. Other methods, with the partial exception of tSVD applied directly to Z, fail to separate the disease groups; tSVD recovers structure with much higher variability than Clorinn. At the factor-level, flashier achieves the lowest oracle-aligned rRMSE on $\hat{L}$ and on the reconstructed shared matrix $\hat{L}{\hat{F}}^{⊤}$, with its default Gaussian and sparse point-normal priors yielding nearly identical curves. Clorinn and tSVD achieve the lowest rRMSE on the factors $\hat{F}$, with the gap from the other methods widening as $h^{2}$ increases. FactorGo and GUIDE sit between these on $\hat{L}$ and $\hat{L}{\hat{F}}^{⊤}$ but degrade on $\hat{F}$ as *P* grows; GLEANR has better median rRMSE on $\hat{L}{\hat{F}}^{⊤}$ compared to Clorinn but shows much higher variability across replicates on every metric. A factorization can be close to the truth after an oracle rotation and per-factor rescaling and still fail to deliver loadings whose native cluster structure is interpretable. The gap between Clorinn and tSVD makes the same point on a smaller scale—their aligned $\text{rRMSE}(\hat{L})$ values are comparable, but their adjusted MI scores differ sharply, showing the statistical benefit of applying tSVD to the denoised low-rank $\hat{X}$ rather than to the noisy input Z.

We explored the effect of model misspecification by specifying the “wrong” number of factors (Fig. S3b, available as supplementary data at *Bioinformatics* online) and the effect of increasing overlap between disease categories by varying the fraction of heritability shared across the three disease categories (Fig. S3c, available as supplementary data at *Bioinformatics* online). Clorinn attains the highest adjusted MI at every shared-heritability fraction from 0.2 to 1.0, and at every true *K* we tested. Except tSVD, again with much higher replicate-to-replicate variance, all other methods sit near zero adjusted MI throughout both panels. On the factor-level RMSE metrics (reported in place of rRMSE here for the reasons noted in Section 2.6), the ordering established in Fig. 2 carries over. The hardest rank setting for every method is K=2. With only two true factors and an estimation budget of ten factors, adjusted MI collapses across the board and Clorinn’s margin narrows. At the other extreme, with K=20 true factors, Clorinn’s adjusted MI is actually highest among the rank-misspecification settings, indicating that under-estimating the true rank (assumed K=10 vs. true K=20) is gentler on structural recovery than over-estimating it (assumed K=10 vs. true K=2). As we increase the shared heritability between the disease groups, it becomes increasingly more difficult for Clorinn to identify the true disease groups, and we observe increasingly higher replicate-to-replicate variability in adjusted MI scores.

### 3.2 Application to 2110 UKB traits

Having demonstrated Clorinn’s performance in simulations, we used it to analyse 2110 real traits from the Pan-UKB data. Our preprocessing followed the procedure in Section 2.7. We constructed a matrix of GWAS *Z* scores at 51 368 LD-pruned SNP variants across 2110 traits. We applied Clorinn on this input matrix to obtain the low-rank matrix $\hat{X}$. For NNM-Sparse, we chose the constraints for *r* and $\ell_{1}$ using cross-validation (see Fig. S4, available as supplementary data at *Bioinformatics* online). We then used tSVD to obtain the top 200 factors and their corresponding loadings.

Our first goal was to analyse how the low-rank matrix captures the shared genetic components. The low-rank matrix $\hat{X}$ explains 57.5% of the total variance of the input Z. We classified the 2110 traits into 30 broad disease categories from their text descriptions, using a publicly available sentence-transformers model (ls-da3m0ns/bge_large_medical). Then we clustered the traits using the estimated loadings from the low-rank matrix $\hat{X}$ using *t*-Distributed Stochastic Neighbor Embedding (tSNE) (van der Maaten and Hinton 2008). We observed that trait clusters obtained from $\hat{X}$ match closely with their text-based unsupervised classification (Fig. 3). The clustering of traits using tSNE is based solely on their genetic associations, while the color-coded classification in the figure is based solely on their text description. Thus, the genetic association matrix contains similar information about the traits as their text descriptions and allows clustering into meaningful categories. Of particular note are the large clusters of dietary intake and mental health traits (see legend in figure). This suggests that granular categorical distinctions of phenotypes are possible even when considering large numbers of traits. However, we emphasize that the distances between the clusters in Fig. 3 have little meaning and should not be over-interpreted (Chari and Pachter 2023).

**Figure 3:**
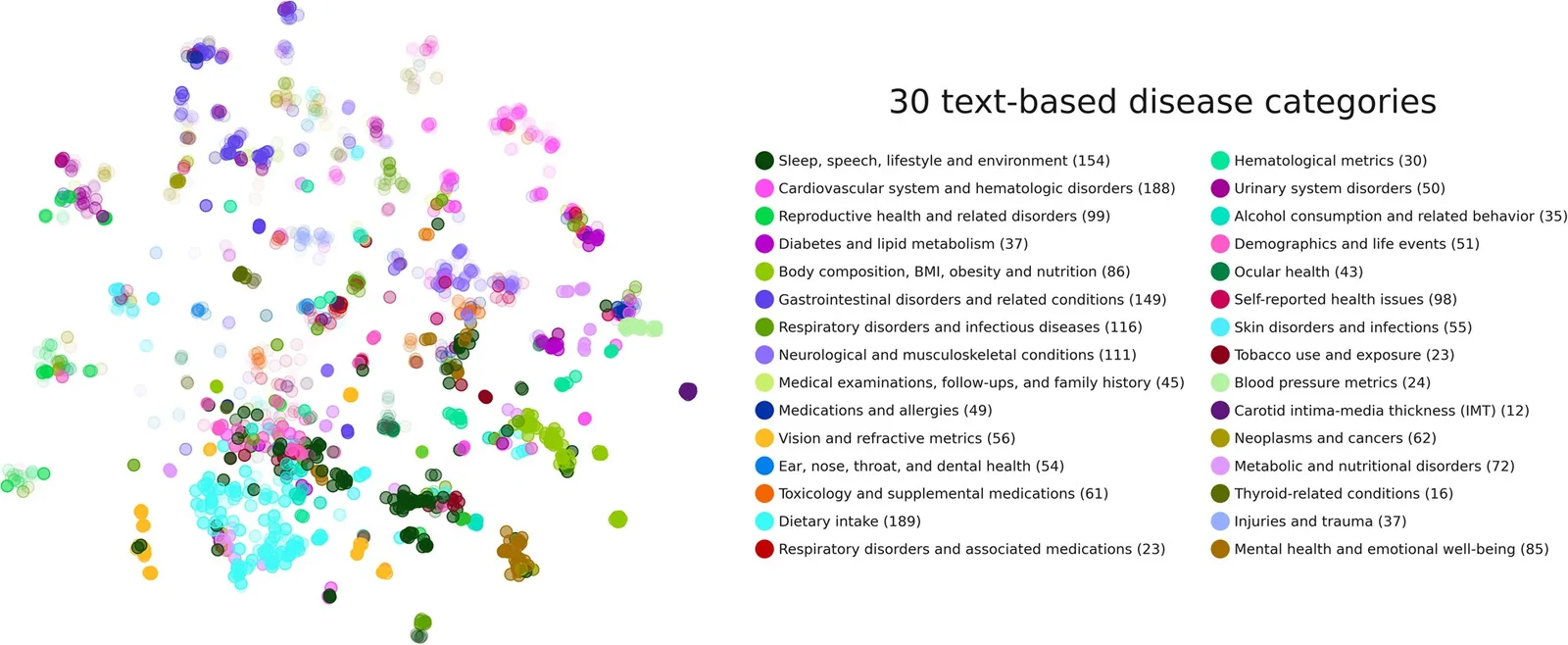
2D tSNE embedding of the Clorinn low rank shared genetic matrix of 2110 PanUKB traits produces clusters in broad agreement with their text descriptions. We classified the 2110 traits into 30 categories from the text descriptions, using a publicly available sentence-transformer model. The number of traits in each category is mentioned in the parentheses. Each point is a trait, colored by its disease category as mentioned in the legend. The opacity of the points increases in proportion to their estimated SNP-heritability.

We next investigated how Clorinn captures the shared genetic components of related traits. We characterized the latent factors $\hat{F}$, their corresponding loadings $\hat{L}$, and how these capture related sets of phenotypes, genes, and variants. We used the top 200 factors for our analyses. These factors explain 88.7% of the variance of $\hat{X}$, see Fig. S5, available as supplementary data at *Bioinformatics* online. We selected type 2 diabetes as a focal trait to explore, given evidence for high heritability from twin studies and population-based tests (Xue *et al.* 2018, Avery and Duncan 2019). We quantified the relative importance of the factors using squared cosine (${\;\text{cos}\;}^{2}$) scores (Abdi and Williams 2010). The top four components of type 2 diabetes (F2, F26, F1, and F18) explained over 55% of the genetic associations (23.6%, 14.4%, 10.7%, and 6.5%, respectively). The scatter plots in Fig. 4a and b show the loadings of all traits corresponding to these factors. The top 20 traits for each factor are annotated on the plots (top 40 traits for F1). Height, weight, and BMI related traits are the major contributors to F1 and F2. F26 and F18 are driven by diabetes-related traits and other major risk factors of type 2 diabetes, including cholesterol levels, indicating that these factors capture the shared genetic components of these phenotypes.

**Figure 4:**
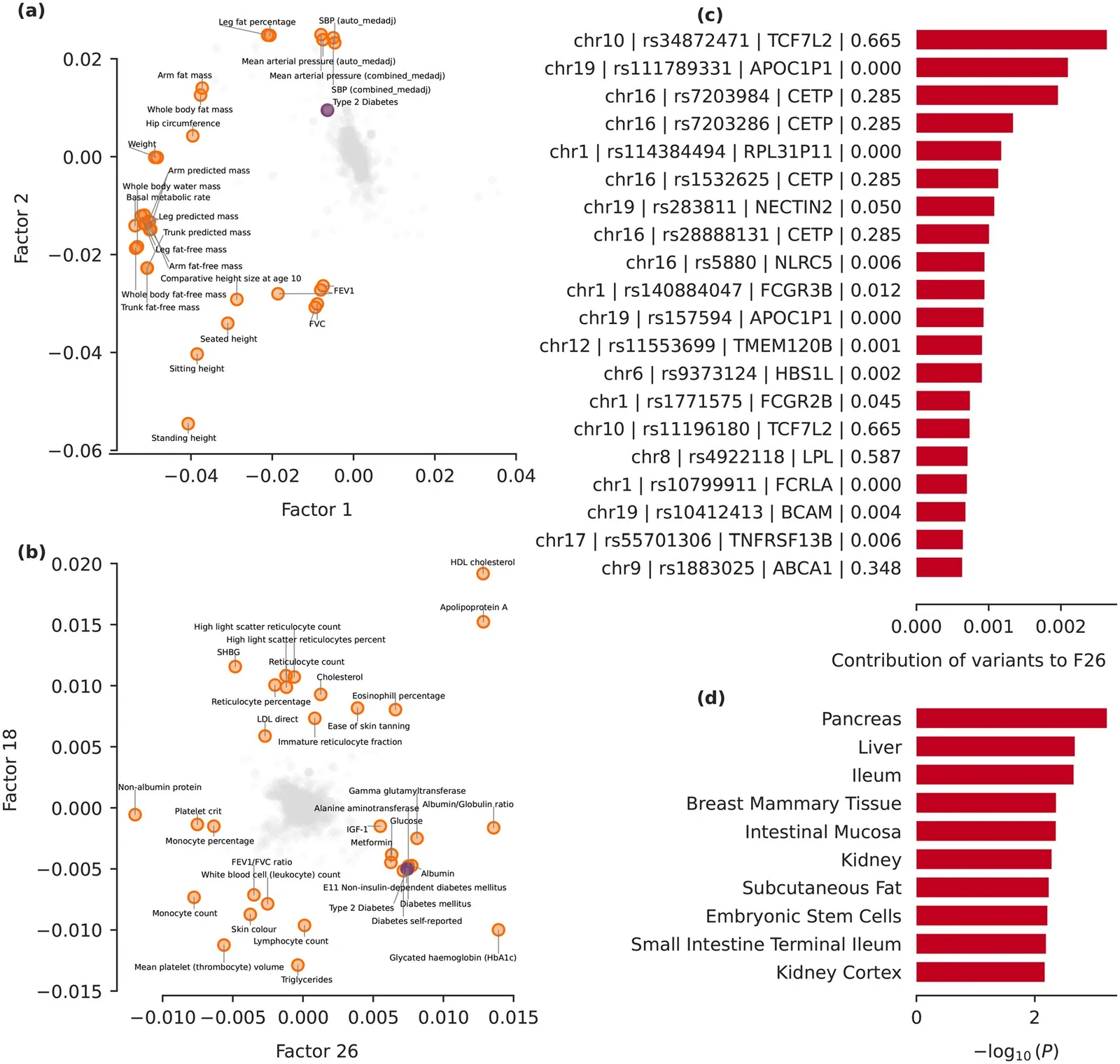
Characterization of hidden factors of type 2 diabetes, as obtained from the PanUKB analysis. (a) Loadings of all traits corresponding to F1 and F2. The top 20–40 traits with highest variation explained are annotated. (b) Same as (a) for F18 and F26. (c) Variant contribution score (20) for F26. Labels on the *y*-axis include chromosome number, rsid of the variant, name of the nearest gene and association score of the gene with type 2 diabetes as obtained from the Open Targets API. (d) S-LDSC annotations for F26, showing the top 10 associations.

Next, we identified the genetic variants (and nearest genes) contributing to F26 using the variant contribution score (Fig. 4). Using the Open Targets API (Buniello *et al*. 2025), we obtained the association score of each gene with type 2 diabetes. Open Targets associations aim to aggregate all evidence relating the target and disease. Association scores range from 0 to 1 with higher scores indicating more confident associations. Among the top 20 variants contributing to F26, several map to genes with high prior T2D association scores (e.g. *TCF7L2*, 0.665; *LPL*, 0.587; *CETP*, 0.285; *NECTIN2*, 0.050; *FCGR3B*, 0.012), thus establishing consistency with previous molecular findings. The top variant for F26 was mapped to *TCF7L2*, which has been previously identified as a significant GWAS association for the condition (del Bosque-Plata *et al.* 2021). Four of the top eight variants for F26 map to *CETP*, a plasma protein responsible for cholesterol transport. Recent research has described the potential of *CETP* inhibitors for the treatment of new-onset diabetes, demonstrating that Clorinn can identify variants with therapeutic potential (Dangas *et al.* 2022). A complementary view of the variants driving F26 is given by the association test of F26 pseudo-*Z* scores per SNP (Fig. S11, available as supplementary data at *Bioinformatics* online). The top hits by association *P*-value broadly overlap with the top hits by contribution score: rs34872471 (*TCF7L2*), rs11196180 (*TCF7L2*), and rs7203984 (*CETP*) appear in both rankings, providing internal consistency between the two summaries.

We can also leverage the fact that traits can have loadings on multiple factors for biological insight. We examined variable traits along F18 and F26, which are the two top factors for type 2 diabetes (Fig. 4b). Several blood traits have large variance, including albumin/globulin ratio and white blood cell leukocyte count. Unsurprisingly, these traits have been previously reported as having significant genetic correlations with diabetes (Howrigan *et al.* 2023). Finally, using stratified-LD score regression (S-LDSC) (Bulik-Sullivan *et al.* 2015, Finucane *et al.* 2015) on 205 tissue- and cell type-specific gene expression annotations, we found that F26 was significantly enriched in pancreas, which is concordant with the known etiology of type 2 diabetes (Macauley *et al.* 2015).

We applied RPCA to derive X and M matrices and repeated the above procedure for comparison against NNM-Sparse in a real-world application (Fig. S8, available as supplementary data at *Bioinformatics* online). We report no overlapping mapped genes between T2D-associated hidden factors across the two optimization algorithms. Additionally, no mapped genes displayed specific contribution patterns, whereby all top variant contribution scores are roughly equal. Further, we find limited overlap between S-LDSC results for enriched tissues, with the top enriched tissue found to be the retina.

### 3.3 Application to 14 psychiatric traits

We sought to compare application of Clorinn to results of a recent cross-disorder analysis of 14 psychiatric traits using gSEM to examine the concordance in latent factors and solution properties (Grotzinger *et al.* 2026). We obtained GWAS summary statistics for each of the 14 analysed traits using the best-powered available study (Table 1). SNPs were clumped using a subset of European individuals from the Haplotype Reference Consortium (HRC) as a reference with a *P*-value threshold of $5\times 10^{-5}$. We excluded SNPs with missing association statistics for ≥4 traits, resulting in a final matrix Z of 14 phenotypes with 5815 SNP effects.

**Table 1:** Table of phenotypes included in the cross-disorder phenotype analysis.^a^

Disorder name	Citation	#SNPs	$h^{2}$ **(%)**	Category
AUD (alcohol-use disorder)	Zhou *et al.* (2023)	536	12.7	Substance use disorders
AN (anorexia nervosa)	Watson *et al.* (2019)	233	11.0	Compulsive disorders
SCZ (schizophrenia)	Trubetskoy *et al.* (2022)	1001	24.0	SCZ/BD
ANX (panic disorder)	Forstner *et al.* (2021)	97	28.0	Internalizing disorders
BD (bipolar disorder)	O’Connell *et al.* (2025)	664	22.0	SCZ/BD
TS (Tourette’s syndrome)	Yu *et al.* (2019)	107	21.0	Compulsive disorders
ASD (autism spectrum disorder)	Grove *et al.* (2019)	166	11.8	Neurodevelopmental disorders
TUD (tobacco-use disorder)	Toikumo *et al.* (2024)	528	5.0	Substance use disorders
CUD (cannabis-use disorder)	Levey *et al.* (2023)	295	6.7	Substance use disorders
PTSD (post-traumatic stress disorder)	Nievergelt et al. (2024)	730	5.3	Internalizing disorders
OCD (obsessive–compulsive disorder)	Strom *et al.* (2024)	80	4.1	Compulsive disorders
MDD (major depressive disorder)	Adams *et al.* (2024)	844	8.4	Internalizing disorders
ADHD (attention-deficit hyperactivity disorder)	Demontis *et al.* (2023)	387	14.0	Neurodevelopmental disorders
OUD (opioid-use disorder)	Deak *et al.* (2022)	223	12.8	Substance use disorders

a #SNPs refers to the number of clumped SNPs in the final pruned matrix contributed by that phenotype, $h^{2}$ estimates are taken from the LDSC parameter estimate from each respective study in European populations on the liability scale (Bulik-Sullivan *et al.* 2015). Phenotype categories are taken from the cross-disorder group analysis (Grotzinger *et al.* 2026).

Applying Clorinn with the NNM-sparse algorithm to Z, we obtained the low-rank matrix X, which explained 18.3% of the total variance of the input Z. We applied PCA to the resultant X matrix and plotted the first two PCs (Fig. 5a). Phenotypes clustered into categories analogous to those described by Grotzinger *et al.* (2026).

**Figure 5:**
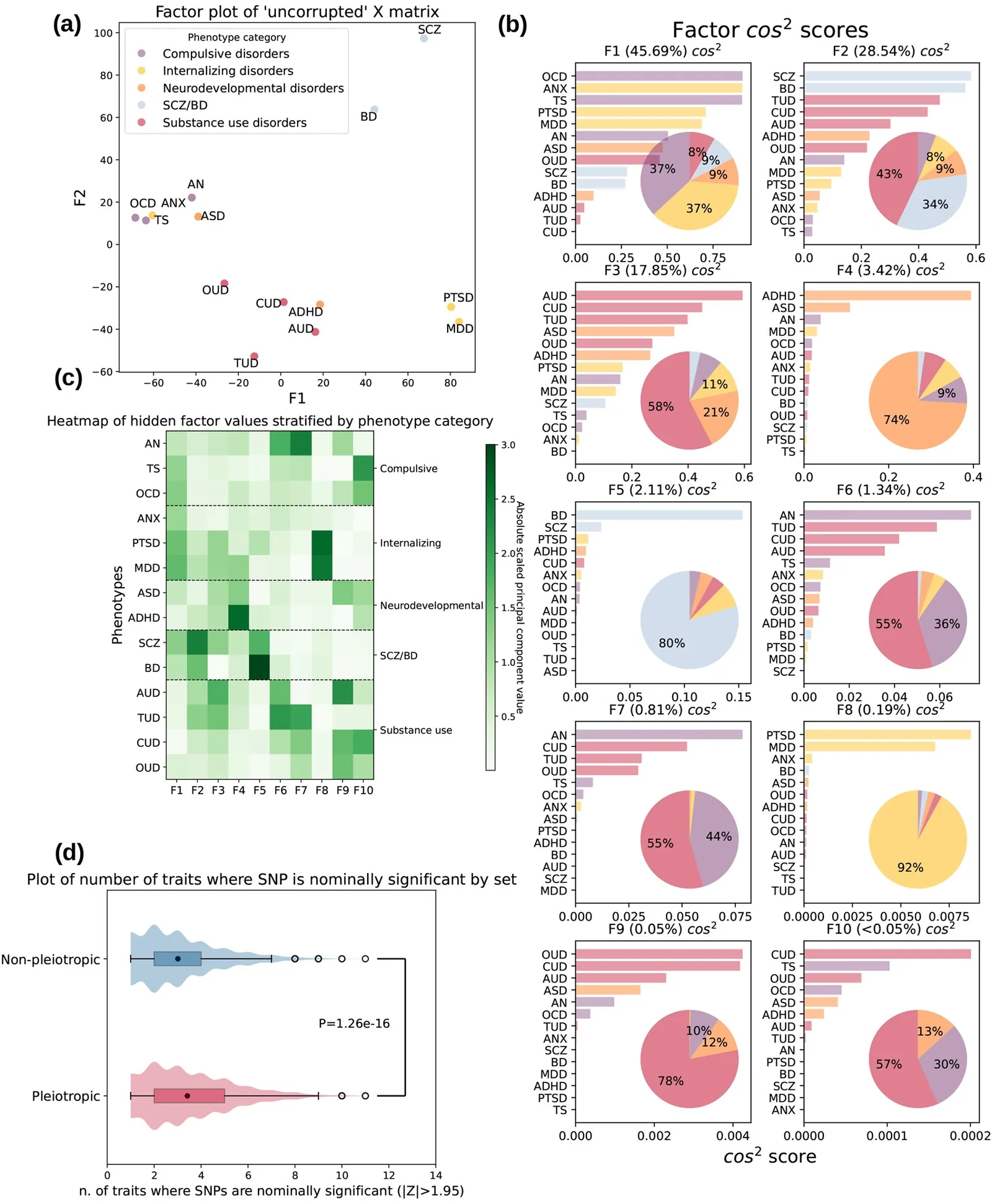
(a) First two factors from PCA of outlier-free X matrix for 14 psychiatric traits. Phenotype categories are derived from the five factor gSEM from Grotzinger *et al*. (2026). Phenotypes are: AUD (alcohol-use disorder), AN (anorexia nervosa), SCZ (schizophrenia), ANX (panic disorder), BD (bipolar disorder), TS (Tourette’s syndrome), ASD (autism spectrum disorder), TUD (tobacco-use disorder), CUD (cannabis-use disorder), PTSD (post-traumatic stress disorder), OCD (obsessive–compulsive disorder), MDD (major-depressive disorder), ADHD (attention-deficit hyperactivity disorder), and OUD (opioid-use disorder). (b) ${\;\text{cos}\;}^{2}$ scores per factor with associated aggregated contribution of phenotype categories in pie charts. Category contribution is measured as the normalized ${\;\text{cos}\;}^{2}$ score of each phenotype to a factor aggregated by phenotype category, ordered from left to right (ascending). The variance explained by each factor is described in parentheses. The color code corresponds to the legend of (a). (c) Factor plot of scaled absolute principal component values across phenotypes. Phenotypes are displayed along the rows, and factor values are along the columns. Values are stratified by phenotypic category along the right side of the *y*-axis. (d) Boxplot of number of traits where SNPs are nominally significant (|Z|≥1.96) across “pleiotropic” and “non-pleiotropic” SNP sets. Pleiotropic SNPs were defined as those with nominally significant loading values on factor 1 (*n* = 3120). The *P*-value was derived from an independent *t*-test of means of number of traits where SNPs are significant.

We derived ${\;\text{cos}\;}^{2}$ scores for the first 10 factors and aggregated contributions by phenotype category to enable comparison with prior gSEM-based latent factor analysis (Grotzinger *et al.* 2026). Every factor from the 5-factor model presented in Grotzinger *et al.* (2026) is represented by at least one factor in our analysis (Fig. 5b). F4 corresponds primarily to neurodevelopmental disorders; F5 to schizophrenia (SCZ)/bipolar disorder (BD) phenotypes; factors 9 and 10 to substance use disorders; F8 to internalizing disorders and F7 to compulsive/substance use disorders. Further, we visualized the absolute scaled activity of hidden factors stratified by phenotype category, offering a more granular view of factor–phenotype specificity (Fig. 5c).

The degree to which principal components load on traits is given by multiplying eigenvectors by the square root of their eigenvalues. A method to conduct statistical hypothesis testing of these factor loadings was previously introduced in Yamamoto *et al.* (2014). Briefly, the correlation between trait values across SNPs and the factor value across SNPs is *t*-distributed under the null hypothesis of no correlation. The intuition is that larger loadings imply stronger correlation of variables to respective factor values. The association of a trait with factor activity can be formally tested by regressing factor values across SNPs on trait values. Following this procedure, we regressed scaled factor values on each SNP individually. We hypothesized that SNPs with significant loadings for the first hidden factor (explaining the largest fraction of phenotypic variance) might have specific properties. We identified 3120 SNPs with statistically significant (nominal *P* < .05) loadings for F1. We expected these SNPs to be nominally significant (*Z* score ≥1.96) in more traits compared to SNPs that do not meet nominal significance thresholds for F1, which are more likely to be disorder-specific. This was indeed the case: disorder-specific SNPs were significant in fewer traits on average than shared SNPs, and this difference was statistically significant (Fig. 5d, $P=1.26\times 10^{-16}$, $P<10^{-3}$ by resampling, see Section 2.8).

Forty-four SNPs were genome-wide significant (GWS, $P<5\times 10^{-8}$) for F1 that were not GWS in any of the included phenotypes, suggesting that Clorinn can adaptively pool statistical evidence across related traits to discover new GWS associations. Three hundred and forty-six SNPs were significant for F1 at a Bonferroni-significant threshold of $P<\frac{0.05}{5815}$. We mapped these SNPs to genes using SNPNexus and tested for enrichment with ShinyGO version 0.82, finding a false discovery rate (FDR)-significant enrichment for arrhythmogenic right ventricular cardiomyopathy ($P=1.9\times 10^{-2}$) based on 216 genes (Ge *et al.* 2020, Oscanoa *et al.* 2020). Further, we used STRING to investigate protein function from these mapped genes, finding FDR-significant associations for various brain-based cellular processes, including neurogenesis, nervous system development, and trans-synaptic signaling (Tables S1 and S2, available as supplementary data at *Bioinformatics* online).

Beyond recapitulating gSEM-defined factors, the F1 association analysis surfaced 44 SNPs reaching genome-wide significance that were not GWS in any single disorder, with MAGMA-mapped genes enriched for synaptic and neurodevelopmental pathways (Fig. S10a, available as supplementary data at *Bioinformatics* online). Further, neuronal projection pathways were significantly enriched in GO cellular components, as well as neuronal midbrain subtype cell signatures. Reported genes queried against GWASCatalog showed overlap with multiple psychiatric risk phenotypes, including cognitive ability, feelings of loneliness, and general risk tolerance (Fig. S10b, available as supplementary data at *Bioinformatics* online). Top MAGMA-associated genes included *KIRREL3, ERBB4*, and *CNTN5* (see Manhattan plot, Fig. S10d, available as supplementary data at *Bioinformatics* online). *KIRREL3* encodes a protein for neuronal cell adhesion, and has been the focus of study for its role in disrupted cognitive processes (Querzani *et al.* 2023). Genetic variation in *ERBB4*, a previously reported risk gene for bipolar disorder and schizophrenia, has been implicated in altered cortical morphology in a pediatric cohort (Douet *et al.* 2015). *CNTN5*, a contactin gene involved in nervous system development, has previously been implicated as a risk gene for psychiatric-associated cognitive phenotypes such as neuroticism (Morris *et al.* 2020).

Our RPCA comparison experiments showed good concordance with the NNM-sparse algorithm in terms of clustering and ${\;\text{cos}\;}^{2}$ scores (Fig. S9, available as supplementary data at *Bioinformatics* online). However, we note that hidden factor composition differs compared to NNM-sparse experiments.

## 4 Discussion

In this work, we introduced Clorinn, a convex framework that decomposes a matrix of GWAS *Z* scores into a low-rank shared component and a sparse contamination component before any factor analysis is performed. The two ingredients—explicit modeling of sparse contamination, and convex optimization with a global optimum—together address the brittleness of classical PCA to outliers and the dependence of variational and EM-based factorizations on initialization. Across simulations spanning a range of polygenic architectures, a biobank-scale analysis of 2110 PanUKB traits, and a focused application to 14 psychiatric disorders, the resulting low-rank matrix $\hat{X}$ supports downstream analyses, e.g. tSVD-based factor recovery, S-LDSC tissue enrichment, and gene-based MAGMA tests that recapitulate established biology and surface candidate signals for follow-up.

The methodological contribution of Clorinn is the decomposition itself, not the particular choice of downstream factorization. We pair $\hat{X}$ with tSVD here, but $\hat{X}$ could equally serve as input to FactorGo (Zhang *et al.* 2023), flashier (Willwerscheid *et al.* 2024), or any pipeline that currently consumes Z directly. Clorinn is a denoising layer beneath the existing factor-analysis ecosystem rather than a competitor to it.

A practical consequence of convexity is that when a new cohort or new traits are added, the updated $\hat{X}$ is the global optimum of a slightly modified convex problem, and its deviation from the previous solution is bounded by the data perturbation. Non-convex factorizations lack this guarantee—even with fixed random seeds, adding a single trait can shift the recovered structure in ways unrelated to the new information. For consortium-level analyses where summary statistics accrue incrementally, this stability is important. A second consequence of the two-component model is that $\hat{M}$ is itself an output. Large entries may correspond to variant-trait pairs that cannot be explained by shared structure. They are candidates for genuinely trait-specific genetic effects, but also for technical artifacts such as allele flips, imputation errors, or meta-analysis inconsistencies. We did not pursue this in the present work, but we anticipate to expand in this direction in the future.

We find that Clorinn recapitulates gSEM results when applied to 14 psychiatric traits. This is interesting given that Clorinn makes use of information from just 5815 SNPs per trait in comparison to gSEM, which fits a model to a genetic covariance matrix of traits using genome-wide information from approximately 1.6 million HapMap3 variants per phenotype (Verhulst *et al.* 2017). Our cos^2^ embeddings, which describe the loading of a factor on traits showed good agreement with results of the 5-factor model from Grotzinger *et al.* (2026) (Fig. 5b). Specifically, all factors from the 5-factor gSEM model are represented in the cos^2^ score embeddings in at least one factor. Fundamentally, Clorinn seeks to derive latent factors using a different subset of genetic information compared to gSEM (which acts on genome-wide estimates of covariance). This exploratory data analysis technique has great potential for identifying correlated sets of traits belonging to broader groupings, especially where the number of traits is large. An advantage of Clorinn from this standpoint is its potential in both biobank-scale factor analysis and more focused hypothesis-driven applications. Comparable methods are limited to the derivation of factors from smaller numbers of phenotypes. We have demonstrated that Clorinn can be applied to thousands of traits.

We also demonstrate the discovery potential of Clorinn in our psychiatric application via statistical hypothesis testing of factor loadings and the definition of pleiotropic and disorder-specific SNP sets. We found that 44 SNPs were GWS after correlating variant *Z* scores to factor activity across phenotypes which were not GWS in any included phenotypes. Further, we report that F1-associated variants mapped to genes with protein products enriched for synaptic function and nervous system development. Our MAGMA results describe significant gene expression enrichment for mapped genes in brain tissues (Fig. S10a, available as supplementary data at *Bioinformatics* online). This is consistent with the intuition that pleiotropic psychiatric SNPs are involved in multiple brain processes. Further, we find GWASCatalog overlap for multiple neurocognitive risk phenotypes (Fig. S10b, available as supplementary data at *Bioinformatics* online), demonstrating that meaningful biological signal can be recovered via our approach. Finally, we find several significant hits in a gene-based test of *P*-values using MAGMA, including *KIRREL3*, *ERBB4*, and *CNTN5* (Fig. S10c, available as supplementary data at *Bioinformatics* online). These results underscore that relevant biological signal can be recovered using our approach. This framework has the potential to obtain proxy genetic associations with unobserved latent variables, which is a conceptually similar goal to approaches such as gSEM. Our approach is an attractive alternative owing to the computational ease of analysis and the sparsity of genetic information required. The definition of disorder-specific SNP sets could also be used to describe properties related to the residual variance per phenotype from gSEM and FactorGo, although an application of this was not explored in this work. While clumping makes use of information from the whole genome, we are left with pseudo-independent genetic signals per disorder. As such, our conceptual aim differs from gSEM in that we model the correlation structure of “pruned” or “pseudo-independent” genetic signal across heterogeneous groups of disorders, rather than an omnigenic consideration of all traits.

Several limitations of the present work suggest directions for refinement. Clorinn does not infer the rank of $\hat{X}$ from the data; the number of retained factors is determined by the singular value spectrum of $\hat{X}$. Methods that report a posterior number of factors (e.g. FactorGo’s ARD prior) offer a more principled treatment, though in practice this often reduces to a thresholding operation. A probabilistic formulation of Clorinn that infers rank without an arbitrary cutoff is a natural extension. We note that the sparse component $\hat{M}$ returned by our method may contain multiple sources of variation; this can include trait-specific signal or errors arising from different forms of noise. Further work is required to investigate how best to interpret and analyze sparse component entries: Developing per-entry diagnostics that distinguish biological signal from technical contamination would convert $\hat{M}$ from a regularization by-product into an actionable output. Within Clorinn itself, RPCA and NNM-Sparse converge on broadly similar factor structures but disagree at the level of top-contributing variants in both the PanUKB and psychiatric applications; the absence of a cross-validation criterion for RPCA’s λ is the likely cause and a target for future work.

While Clorinn has a direct SNP-mapping through the outlier-free $\hat{X}$, this abstraction may make it more difficult to interpret associated factors and their constituent elements: a denoised *Z* score is neither an observed association statistic nor a posterior estimate of a true effect. An important point of future research will be developing a thorough interpretation of SNP $\hat{X}$ values relative to the input SNP-trait associations. Finally, it has recently been shown that assortative mating inflates estimates of genetic correlation (Border *et al.* 2022). Factorization approaches like Clorinn inherit this inflation, and it remains an open question how such analyses might be appropriately corrected.

Here, we have developed a flexible modeling approach to describe latent genetic factors of an arbitrary number of GWAS results. Our method is reproducible and derives an outlier-free matrix of trait associations before factor discovery, which has several attractive properties. We anticipate that Clorinn can be used for analysis at the trait and variant level in a variety of applications, especially as the number of available phenotypes increases.

## Supplementary Material

btag670_Supplementary_Data

## Data Availability

Our software is publicly available at https://github.com/daklab/clorinn. Simulations are available at https://github.com/banskt/lrma-dsc. Summary statistics from the Pan-UK Biobank and psychiatric GWAS used in this study were obtained from publicly available sources as referenced in the Section 2. Derived data including low-rank matrices and factor loadings are available upon request.
