# Supplementary material for: Convex approaches to isolate the shared and distinct genetic components of complex traits
Source: Bioinformatics. 2026 Sep 17;42(10):btag670. doi: 10.1093/bioinformatics/btag670 (PMC13633666; doi:10.1093/bioinformatics/btag670)
Supplement: Supplementary file 1 [file btag670_supplementary_data.zip › clorinn_manuscript_supplementary.pdf]

Supplementary material for Convex approaches to isolate the shared and distinct genetic components of complex traits

Saikat Banerjee,[1,†] **Shane O'Connell**,[2,3,4,5,†] **Sarah M.C. Colbert**,[2,3,4,5] **Niamh Mullins**[2,3,4,5] **and David A. Knowles**[1,6,7,*]

[1]New York Genome Center, 101 6th Ave, New York, 10013, NY, USA

[2]Department of Psychiatry, Icahn School of Medicine at Mount Sinai, 1 Gustave L. Levy Place, New York, 10029, NY, USA

[3]Department of Genetics and Genomic Sciences, Icahn School of Medicine at Mount Sinai, 1 Gustave L. Levy Place, New York, 10029, NY, USA

[4]Charles Bronfman Institute for Personalized Medicine, Icahn School of Medicine at Mount Sinai, 1 Gustave L. Levy Place, New York, 10029, NY, USA

[5]Department of Artificial Intelligence and Human Health, Icahn School of Medicine at Mount Sinai, 1 Gustave L. Levy Place, New York, 10029, NY, USA

[6]Department of Computer Science, Columbia University, 500 West 120 Street, New York, 10027, NY, USA

[7]Department of Systems Biology, Columbia University, 1130 St. Nicholas Avenue, New York, 10032, NY, USA

[†]Contributed equally.[*]Corresponding author. dak2173@columbia.edu

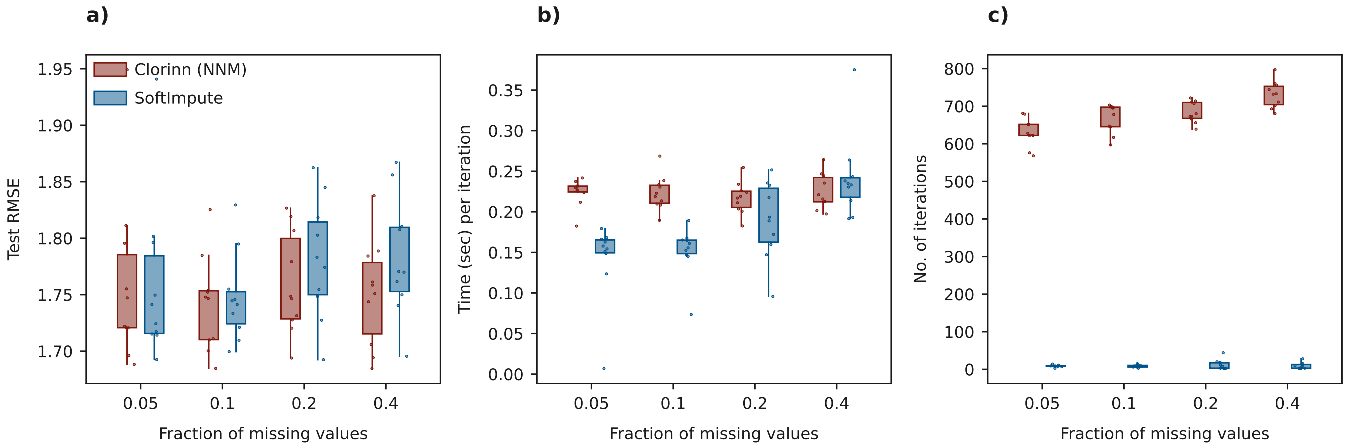

Figure S1 Comparison of Frank-Wolfe algorithm with softImpute algorithm. We hold out a certain fraction of input data (shown on the x-axis of each panel), and use different algorithms to estimate the held-out test data. We perform 5-fold cross-validation on the training data (without observing the held-out test data) to estimate the threshold parameters for the different methods and report the results using the optimum parameters (as determined by the cross-validation). (a) RMSE of the held-out test data estimated by different methods. (b) Average computational time (in seconds) per iteration of the different methods, and (c) Number of iterations required for convergence.

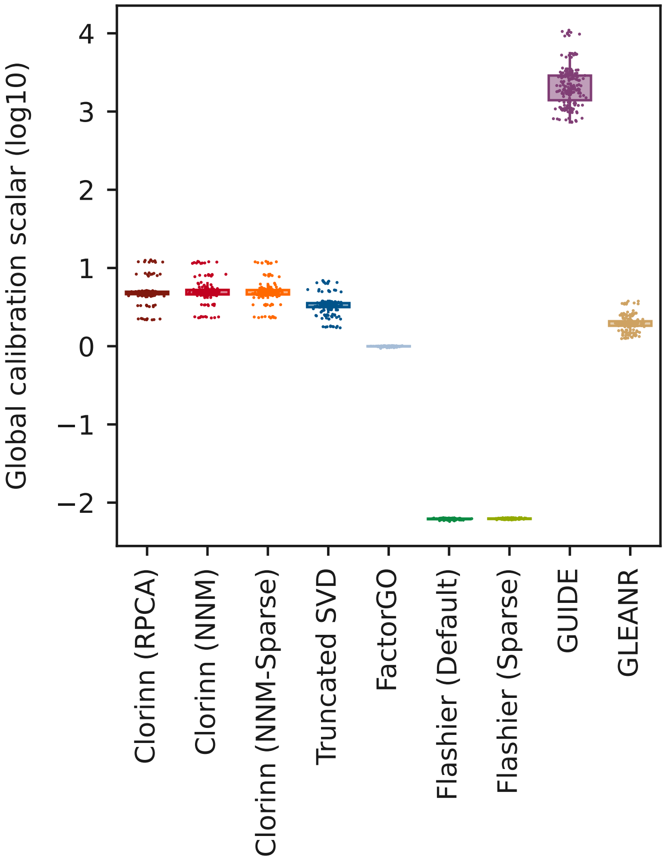

Figure S2 Comparison of global amplitude calibration of competing methods. The global amplitude scalar $\widehat{s} = \arg\min_s \|\mathbf{X} - s\widehat{\mathbf{X}}\|_F^2$ is the least-squares scalar that brings each method's reconstructed $\widehat{\mathbf{X}}$ onto the scale of the ground-truth. $\log_{10}(\widehat{s}) = 0$ means the two are already on the same scale. Positive (or negative) values indicate attenuation (or amplification) – the estimate must be scaled up (or down) to match the truth. Magnitudes of 1 and 2 correspond to multiplicative corrections of $10\times$ and $100\times$, respectively. Each point is one simulation replicate; replicates are pooled across all parameter settings. The spread between methods reflects differences in the *scale convention* at which each reports $\widehat{\mathbf{L}}\widehat{\mathbf{F}}^\top$, not differences in structural recovery, motivating the global calibration step.

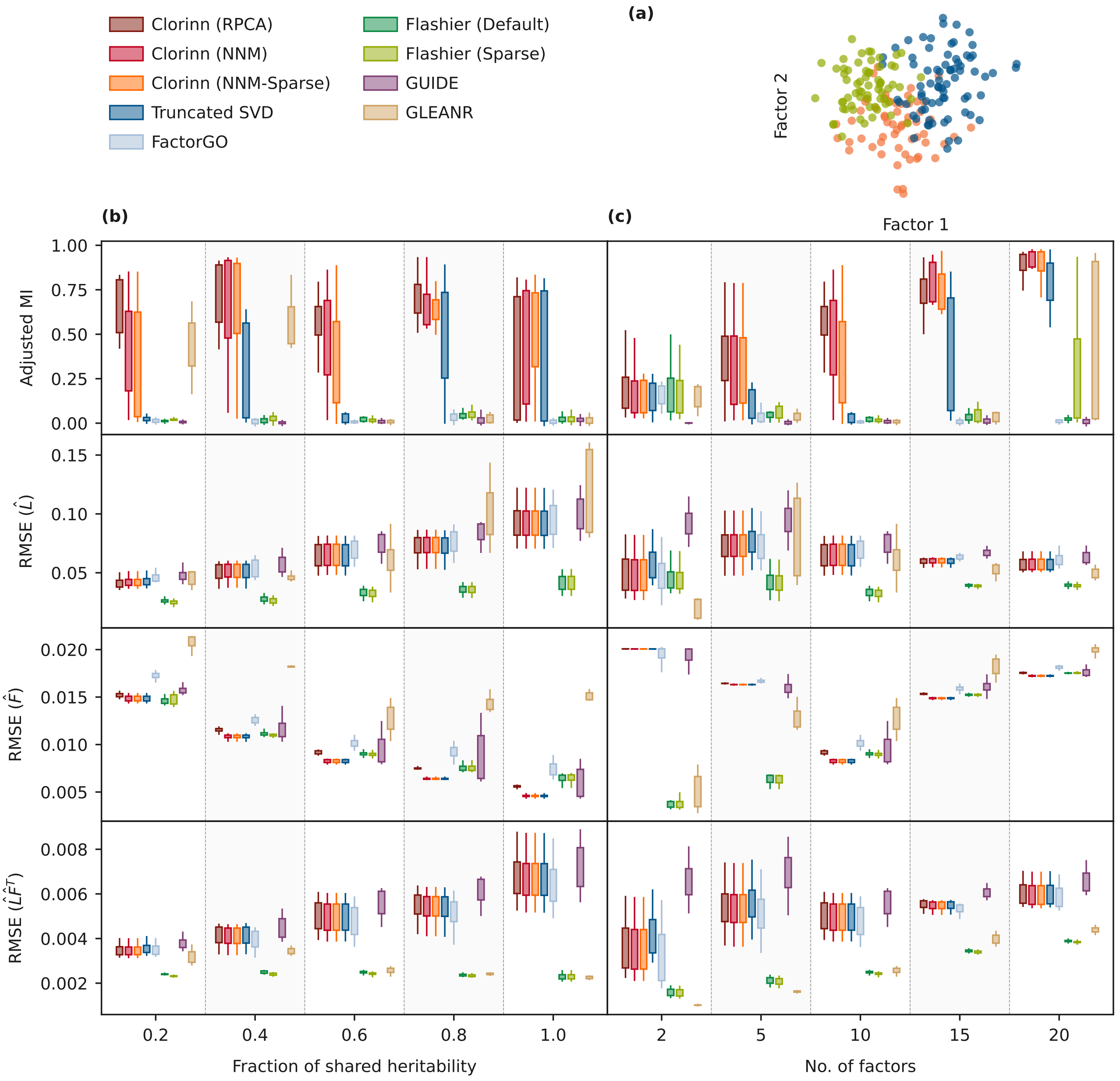

Figure S3 (a) Loadings of the first two factors in our ground truth in one arbitrarily chosen simulation replicate (200 traits simulations from 3 disease categories indicated by different colors). (b) Comparison of methods as we vary the fraction of heritability shared by the three broad disease categories, with the remaining parameters held at their default values. Each row reports one recovery metric, and each boxplot summarizes 20 simulation replicates. *Adjusted mutual information* (MI) is obtained by hierarchical clustering of each method's *native* estimated loadings $\widehat{\mathbf{L}}$ (no calibration or alignment) and measures whether the recovered partition matches the true disease categories. $\mathrm{rRMSE}(\widehat{\mathbf{L}})$ and $\mathrm{rRMSE}(\widehat{\mathbf{F}})$ were obtained after global amplitude calibration and Procrustes alignment with per-factor rescaling against the ground truth. $\mathrm{rRMSE}(\widehat{\mathbf{L}}\widehat{\mathbf{F}}^\top)$ is the relative RMSE of the reconstructed low-rank signal $\widehat{\mathbf{X}} = \widehat{\mathbf{L}}\widehat{\mathbf{F}}^\top$ after global calibration; the alignment step is product-preserving and does not affect this metric. (c) Same as (b) but with varying number of ground truth hidden factors, K. Each method used $K = 10$ for estimating the hidden factors irrespective of the ground truth.

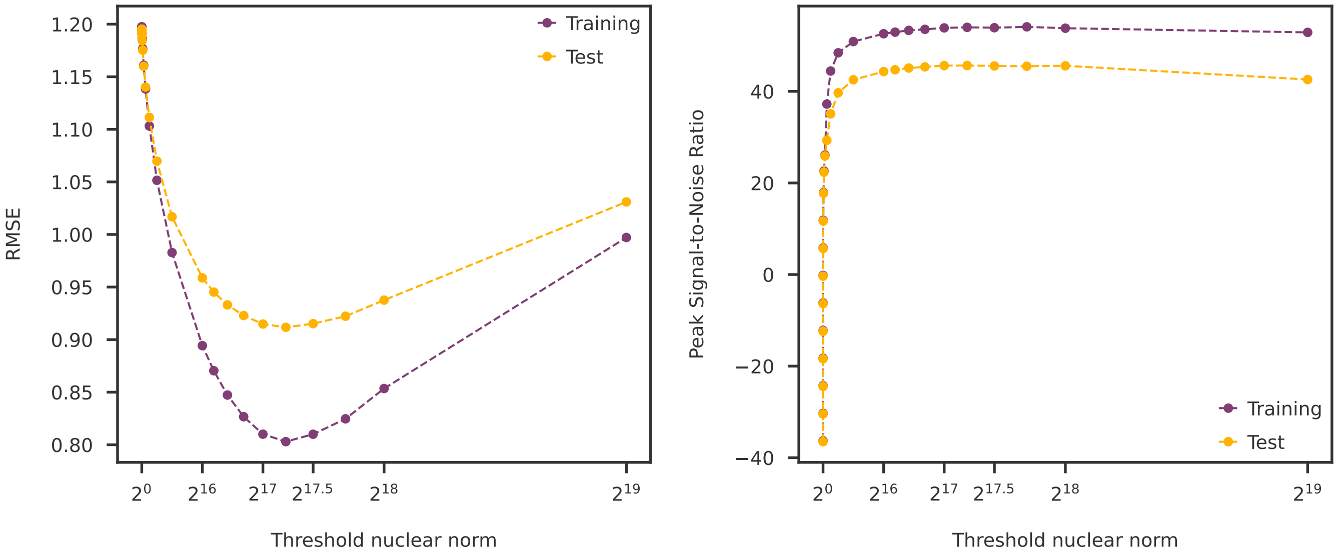

Figure S4 Cross-validation to obtain rank threshold for NNM-Sparse FW algorithm on PanUKB data. Both error metrics, root mean square error (RMSE, left panel) and peak signal-to-noise ratio (right panel) suggests a threshold of $r = 2^{17.25} = 155872$.

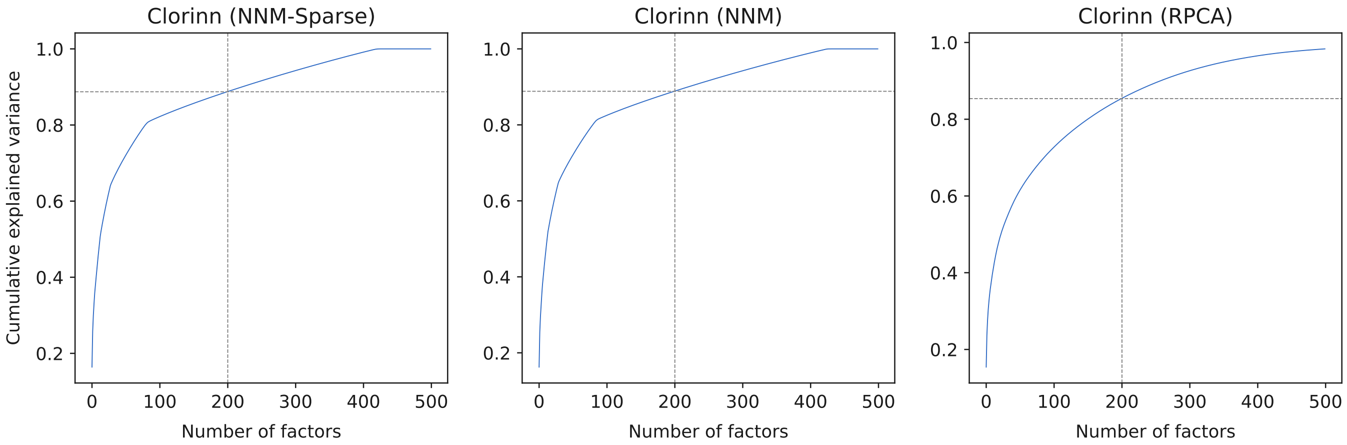

Figure S5 Cumulative explained variance by the singular values obtained from the PCA of the low rank matrix $\widehat{\mathbf{X}}$ estimated from the PanUKB data. The three panels show the different LRMA methods implemented in Clorinn.

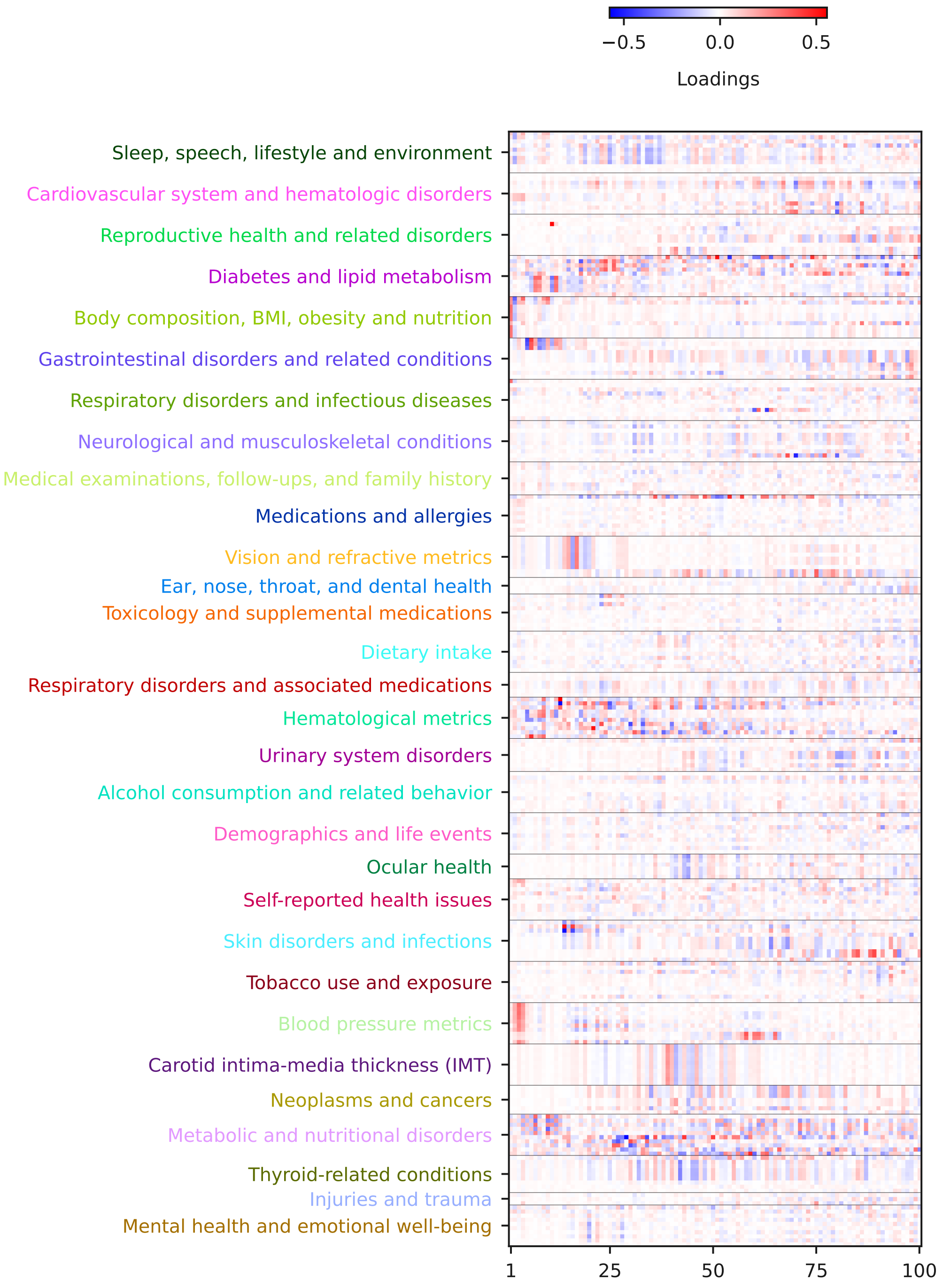

Figure S6 Contribution of some selected traits on the top 100 factors (shown on x-axis) obtained from the application of Clorinn on the PanUKB dataset. The heatmap shows the magnitude of the loadings (color scale shown on the top). The traits were selected based on their estimated heritability ($h^2 \geq 0.01$), and a maximum of 10 traits were retained for each text-based disease category, see Figure 3 in main text.

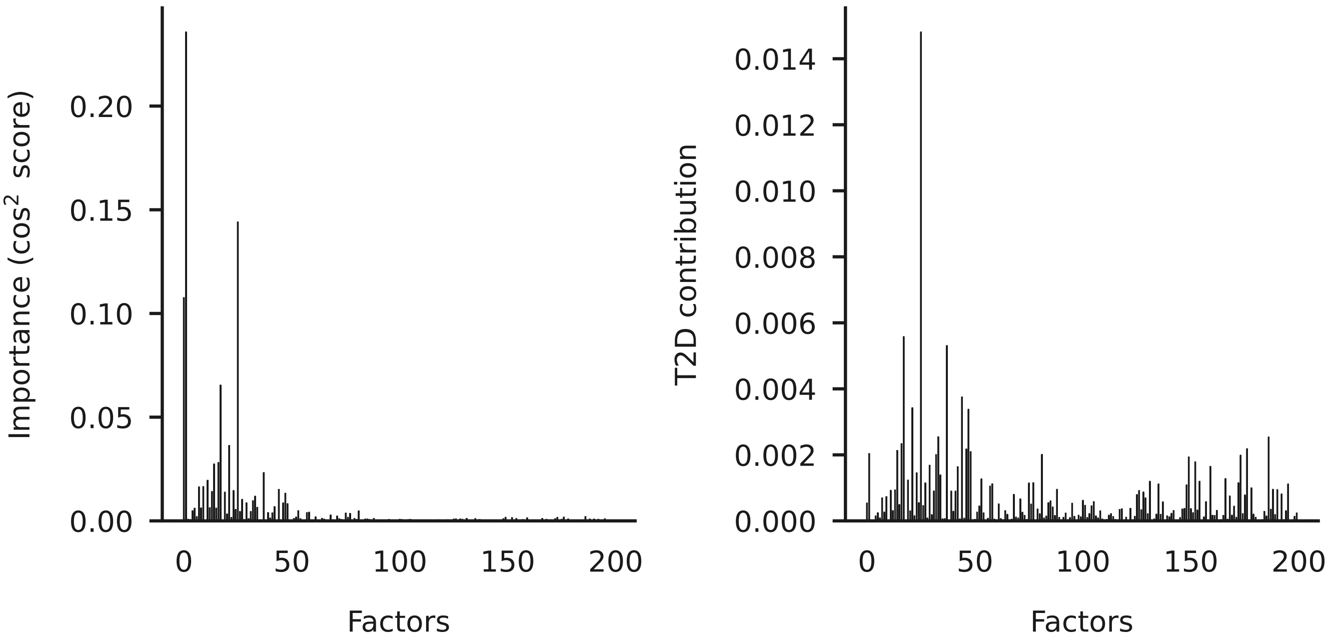

Figure S7 Importance of hidden factors on Type 2 diabetes, obtained by applying Clorinn NNM-Sparse on the PanUKB data. The left panel shows the importance of the factors as measured by the $\cos^2$ and the right panel shows the contribution score of T2D on the different factors.

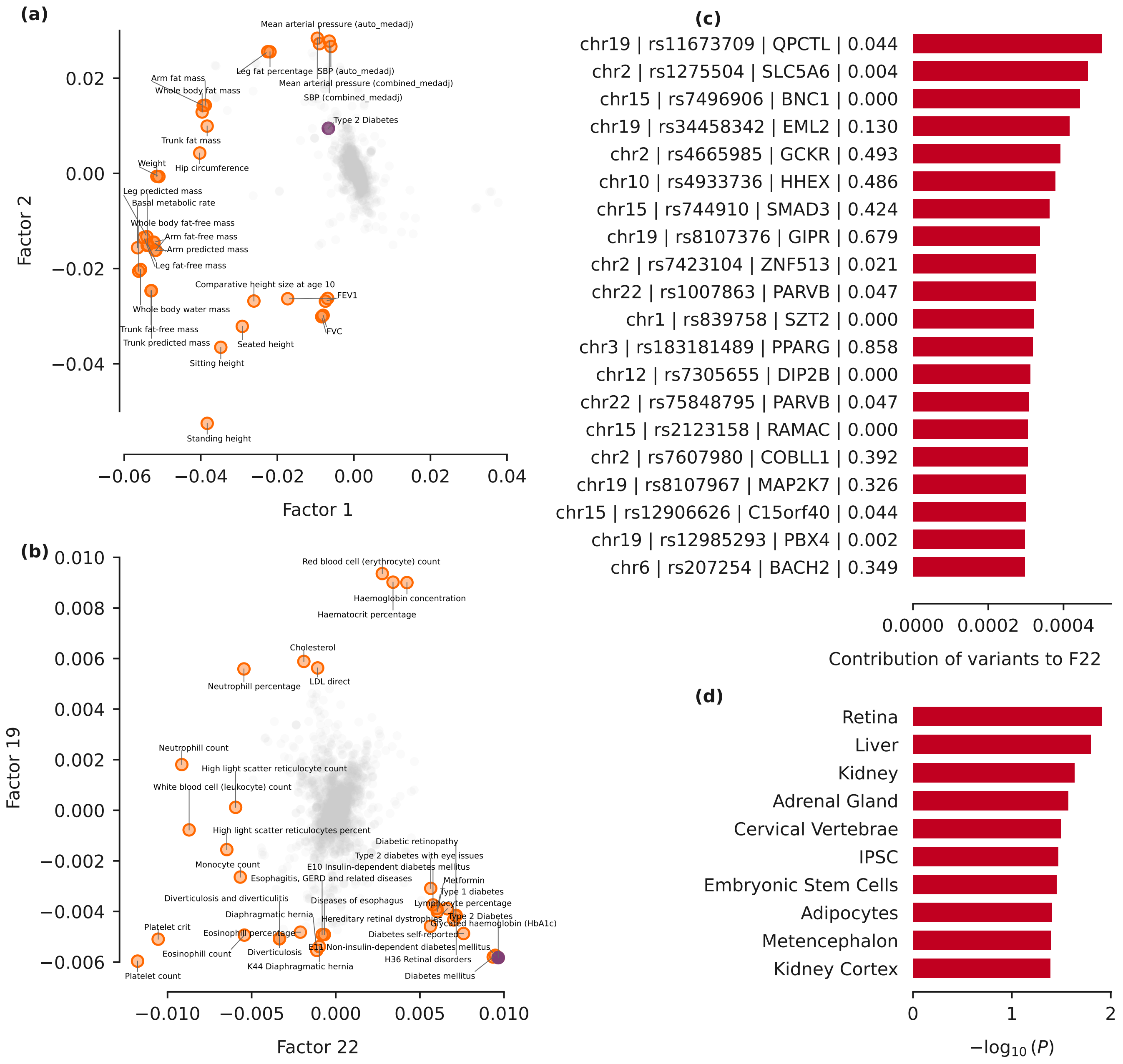

Figure S8 Characterization of hidden factors of Type 2 diabetes, as obtained from Robust PCA on the PanUKB data. (a) Loadings of all traits corresponding to F1 and F2, traits with highest variation are annotated. (b) Same as (a) for F19 and F22. (c) Contribution score of variants to F22. Labels on the y-axis include chromosome number, rsid of the variant, name of the nearest gene and association score of the gene with Type 2 diabetes as obtained from the Open Targets API. (d) Significantly enriched LDSC-SEG annotations for F22.

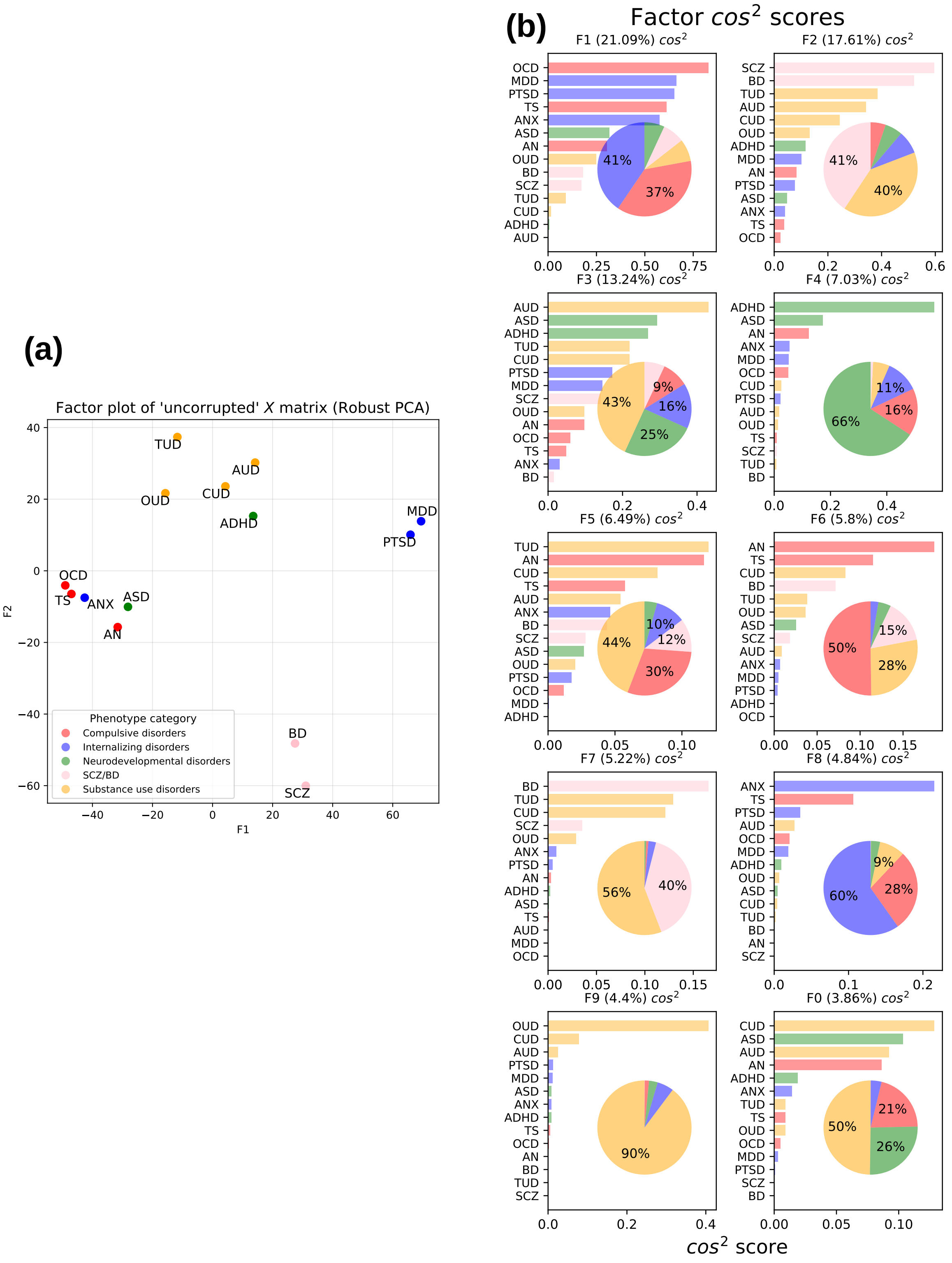

Figure S9 Robust PCA application to data from 14 psychiatric traits. (a) PCA plot of low rank component from Robust PCA. (b) Squared cosine values aggregated across phenotype categories; legend labels are inherited from (a).

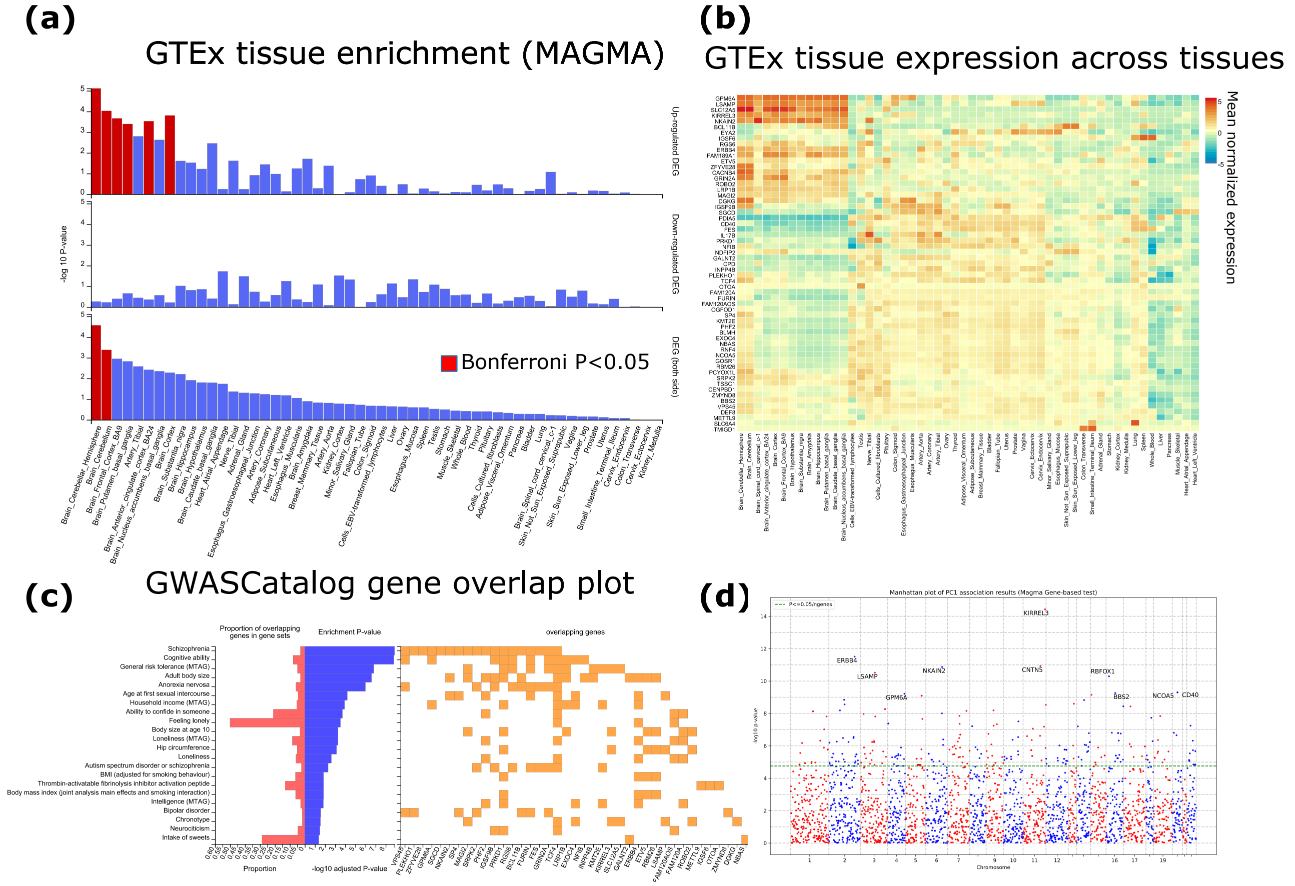

Figure S10 Results from MAGMA analyses using FUMA for association test statistics for PC1 for 14 psychiatric conditions. (a) GTEx tissue enrichment results. The $-\log_{10}$ p-value of enrichment is displayed on the y-axis, and the tissues are displayed on the x-axis. Red entries denote tissues where enrichment p-values are less than the Bonferroni-correct significance threshold. (b) Heatmap of genes mapped to significant independent hits, where tissues are clustered along the x-axis and genes are clustered along the y-axis. Values represent the average normalized gene expression per tissue (zero-mean). (c) GWASCatalog results for genes mapped to significant independent hits. Phenotypes are displayed along the y-axis, and proportion of overlap against the overall set, $-\log_{10}$ adjusted p-value, and genes are displayed along the x-axis. (d) Manhattan plot of gene-based MAGMA association test using association test statistics for PC1/F1. Significance is described as $0.05/n_g$, where n_g is the number of genes tested. The top 10 genes ranked by adjusted p-value are annotated.

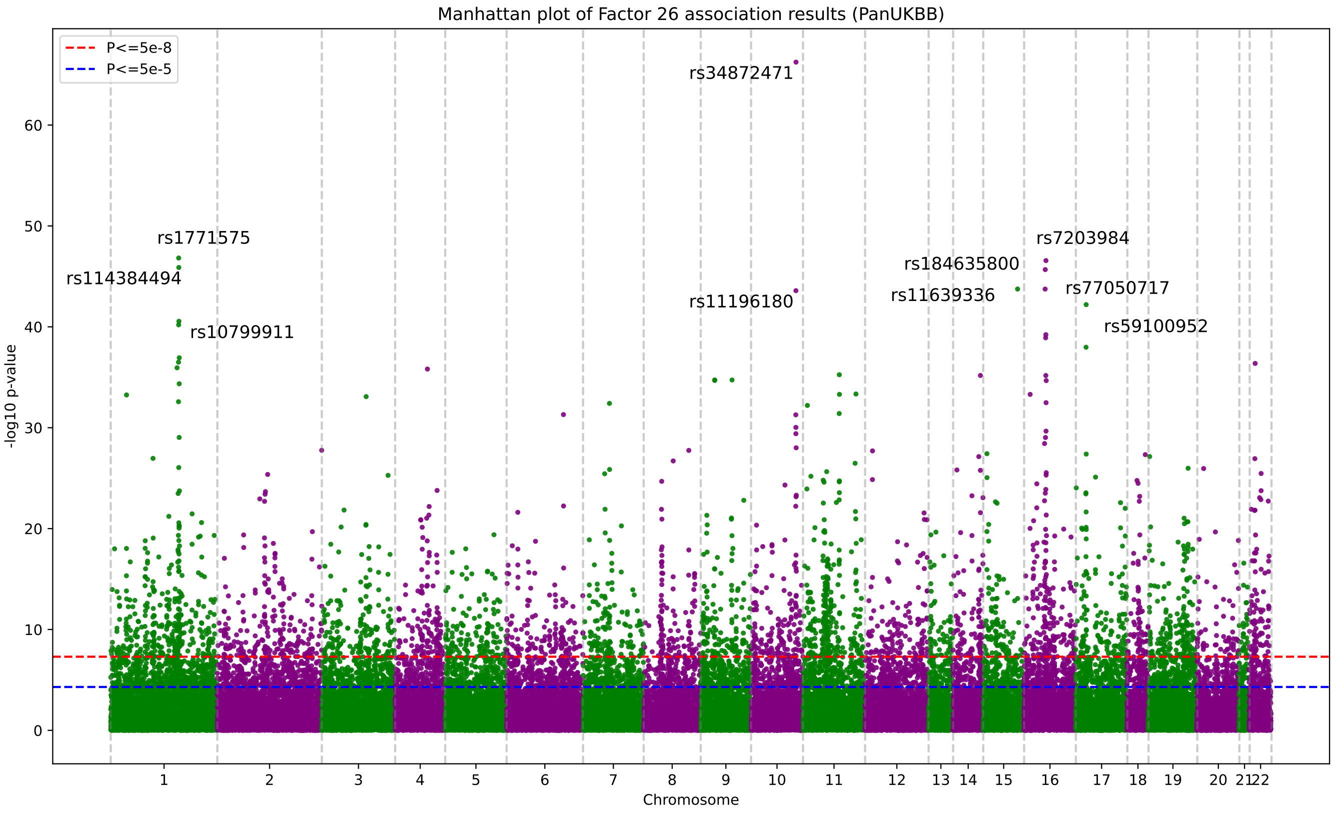

Figure S11 Manhattan plot of Factor 26 association test statistics of pseudo-Z scores. The top 10 SNPs ordered by $-log_{10}$ p-value are annotated. Significance thresholds are denoted by a red line (genome-wide significance) and a blue line (suggestive significance).
